# How *Caenorhabditis elegans* Senses Mechanical Stress, Temperature, and Other Physical Stimuli

**DOI:** 10.1534/genetics.118.300241

**Published:** 2019-03-11

**Authors:** Miriam B. Goodman, Piali Sengupta

**Affiliations:** * Department of Molecular and Cellular Physiology, Stanford University, California 94304 and; † Department of Biology, Brandeis University, Waltham, Massachusetts 02454

**Keywords:** *C. elegans*, mechanosensation, thermosensation, WormBook

## Abstract

*Caenorhabditis elegans* lives in a complex habitat in which they routinely experience large fluctuations in temperature, and encounter physical obstacles that vary in size and composition. Their habitat is shared by other nematodes, by beneficial and harmful bacteria, and nematode-trapping fungi. Not surprisingly, these nematodes can detect and discriminate among diverse environmental cues, and exhibit sensory-evoked behaviors that are readily quantifiable in the laboratory at high resolution. Their ability to perform these behaviors depends on <100 sensory neurons, and this compact sensory nervous system together with powerful molecular genetic tools has allowed individual neuron types to be linked to specific sensory responses. Here, we describe the sensory neurons and molecules that enable *C. elegans* to sense and respond to physical stimuli. We focus primarily on the pathways that allow sensation of mechanical and thermal stimuli, and briefly consider this animal’s ability to sense magnetic and electrical fields, light, and relative humidity. As the study of sensory transduction is critically dependent upon the techniques for stimulus delivery, we also include a section on appropriate laboratory methods for such studies. This chapter summarizes current knowledge about the sensitivity and response dynamics of individual classes of *C. elegans* mechano- and thermosensory neurons from *in vivo* calcium imaging and whole-cell patch-clamp electrophysiology studies. We also describe the roles of conserved molecules and signaling pathways in mediating the remarkably sensitive responses of these nematodes to mechanical and thermal cues. These studies have shown that the protein partners that form mechanotransduction channels are drawn from multiple superfamilies of ion channel proteins, and that signal transduction pathways responsible for temperature sensing in *C. elegans* share many features with those responsible for phototransduction in vertebrates.

THE ability of animals to detect mechanical, thermal, and other physical stimuli is conserved across phyla and plays a key role in their navigation of variable and harsh environmental conditions. These senses enable animals to mate, find food, and avoid danger, and depend on the functions of neurons specialized to detect these stimuli. Transduction of these stimuli in sensory neurons is mediated via signaling pathways that converge on ion channels, thereby converting physical stimuli into electrical signals that propagate through the nervous system to trigger appropriate behavioral responses. Animals use diverse receptors and signaling pathways to reliably sense and respond to physical cues. An intriguing feature of sensory transduction is the convergent and divergent evolution of sensory molecules and mechanisms allowing optimization of animal survival in specialized ecological niches. For instance, a signaling pathway used in a sensory modality in one species may evolve to detect a different cue in another. Similarly, the same cue may be sensed via distinct mechanisms in different species. Thus, a complete understanding of mechano- and thermosensation requires identification and comparison of transduction mechanisms across animals.

This chapter describes the current state of knowledge regarding the transduction of mechanical and thermal cues in *Caenorhabditis elegans* nematodes. Research on this topic is enabled by the ability of researchers to combine genetic discovery with quantitative analyses of behaviors and physiological measurements of sensory neuron responses in living animals. Neuroanatomical studies presciently predicted the identities of mechanosensory and thermosensory neurons, which were confirmed by cell ablation followed by behavioral analyses (Chalfie *et al.* 1985; Mori and Ohshima 1995). Genetic analyses that identified mutants with sensory defects have now provided detailed molecular insights into sensory signal transduction mechanisms employed by individual sensory neuron types in this organism. The development of genetically encoded sensors for calcium and other second messengers, and of techniques for electrophysiology, have also now allowed studies of sensory neuron physiology in living animals. Findings in *C. elegans* have contributed several pivotal concepts to the broader field of sensory transduction. Analyses in *C. elegans* revealed that even simple animals rely on a collection of specialized sensory neurons for mechanosensory transduction, and that multiple classes of ion channels have been harnessed to subserve this function. Paradigms for linking mechanosensory transduction to the action of specific ion channels via mutations that alter ion selectivity were established in *C. elegans* and have been adopted in other systems, including the hair cells responsible for hearing and balance in mammals. Analyses of thermosensation in *C. elegans* have provided insights into the molecular mechanisms that enable an organism to respond sensitively and robustly to a cue over a wide dynamic range in an experience-dependent manner, and have described principles that are broadly applicable to diverse sensory systems.

Here, we focus primarily on the neurons, molecules, and signaling pathways used by *C. elegans* to detect mechanical and thermal stimuli and briefly describes the neurons and molecules known to mediate responses to magnetic and electrical fields, light, and humidity. Laboratory methods for delivering physical stimuli to *C. elegans* nematodes are presented as an Appendix. The molecular events that give rise to chemosensation of soluble and volatile attractants and repellents, of gases, and of pheromones, are discussed in an upcoming Wormbook chapter.

## Sensory Anatomy of *C. elegans* Nematodes

**Figure 1:**
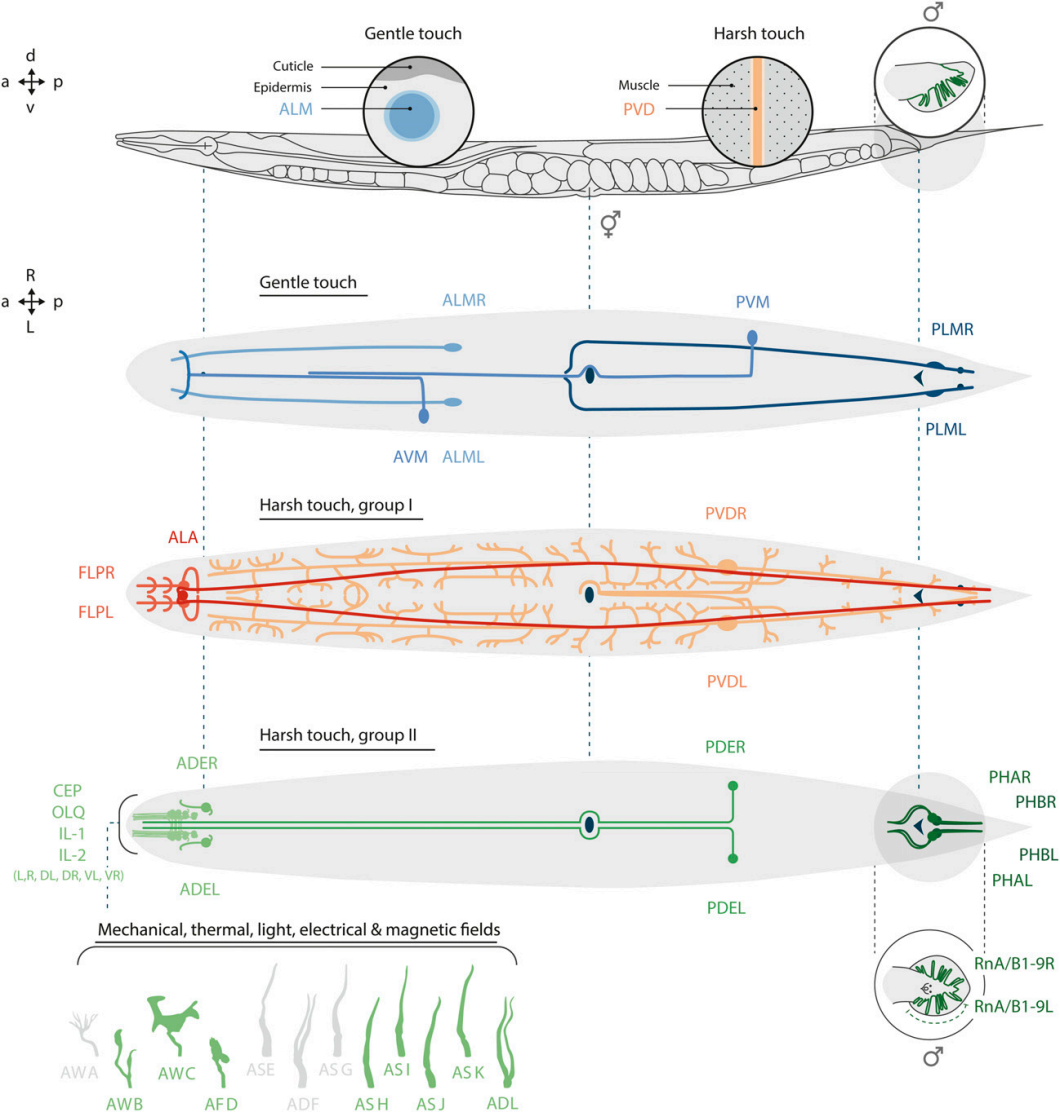
Positions of sensory neurons in adult *C. elegans*. (Top) Lateral view of an adult hermaphrodite and male tail. Insets illustrate the association of gentle touch receptor neurons (TRNs; only ALM is shown) and the multidendritic nociceptive neurons (PVD) with the epidermis and muscle, respectively. (Middle two panels) Inside view of the six TRNs (ALML/R, AVM, PVM, PLML/R), the dendritic arbors of the multidendritic FLP and PVD neurons and the ALA harsh touch receptor. (Bottom) Positions of ciliated sensory neurons in hermaphrodites and in the male tail. Inset shows the shapes of the ciliated endings that terminate in the amphid sensilla. Ciliated neurons indicated in green are discussed in this chapter; those indicated in gray will be discussed in a forthcoming Wormbook chapter on chemical sensing.

Despite their compact nervous system, *C. elegans* nematodes have at least 70 neurons predicted to function as sensory neurons (Ward *et al.* 1975; Perkins *et al.* 1986; White *et al.* 1986; Hall *et al.* 2006). These neurons (Figure 1 and Table 1) detect soluble and volatile chemicals, gases, mechanical stress, temperature, light, and additional sensory cues. Sixty of the sensory neurons in the adult hermaphrodite, and an additional 52 neurons in the adult male, contain primary cilia at their sensory endings (Ward *et al.* 1975; Perkins *et al.* 1986; Doroquez *et al.* 2014). These cilia are microtubule-based structures that are structurally specialized for the unique functions of each sensory neuron type, and house all primary sensory signal transduction molecules. A subset of nonciliated sensory neurons including PVD and FLP exhibits highly complex dendritic morphologies that also shape the functions of these neurons (Halevi *et al.* 2002; Oren-Suissa *et al.* 2010; Smith *et al.* 2010; Albeg *et al.* 2011) (Figure 1). Readers are referred to the chapter by Leroux and Buelow (Wormbook) for details regarding the formation, maintenance, and function of sensory dendrites and cilia.

**Table 1:** Primary sensory neurons mediating responses to physical stimuli in *C. elegans* hermaphrodites

Neuron class (#)	Physical stimuli sensed*^a^*	WormAtlas link
*Anterior amphid sensilla*		
AWC (2)	Physiological temperature, noxious heat, electrical field	http://wormatlas.org/neurons/Individual%20Neurons/AWCframeset.html
AFD (2)	Physiological temperature, noxious heat, magnetic field, humidity	http://wormatlas.org/neurons/Individual%20Neurons/AFDframeset.html
ASH (2)	Harsh touch, electrical field, light	http://wormatlas.org/neurons/Individual%20Neurons/ASHframeset.html
ASI (2)	Physiological temperature	http://wormatlas.org/neurons/Individual%20Neurons/ASIframeset.html
ASJ (2)	Physiological temperature, electrical field, light	http://wormatlas.org/neurons/Individual%20Neurons/ASJframeset.html
*Anterior inner labial sensilla*		
IL1 (6)	Harsh touch	http://wormatlas.org/neurons/Individual%20Neurons/IL1frameset.html
IL2 (6)	Harsh touch	http://wormatlas.org/neurons/Individual%20Neurons/IL2frameset.html
*Anterior outer labial sensilla*		
OLQ (4)	Harsh touch	http://wormatlas.org/neurons/Individual%20Neurons/OLQframeset.html
*Anterior deirid sensilla*		
ADE (2)	Harsh touch, texture	http://wormatlas.org/neurons/Individual%20Neurons/ADEframeset.html
*Anterior cephalic sensilla*		
CEP (4)	Harsh touch, texture	http://wormatlas.org/neurons/Individual%20Neurons/CEPframeset.html
*Anterior body (not associated with sensilla)*		
ALM (2)	Gentle touch	http://wormatlas.org/neurons/Individual%20Neurons/ALMframeset.html
AVM (1)	Gentle touch	http://wormatlas.org/neurons/Individual%20Neurons/AVMframeset.html
AQR (1)	Harsh touch	http://wormatlas.org/neurons/Individual%20Neurons/AQRframeset.html
BDU (2)	Harsh touch	http://wormatlas.org/neurons/Individual%20Neurons/BDUframeset.html
FLP (2)	Harsh touch, noxious heat, humidity	http://wormatlas.org/neurons/Individual%20Neurons/FLPframeset.html
*Posterior phasmid sensilla*		
PHA (2)	Harsh touch	http://wormatlas.org/neurons/Individual%20Neurons/PHAframeset.html
PHB (2)	Harsh touch	http://wormatlas.org/neurons/Individual%20Neurons/PHBframeset.html
*Posterior deirid sensilla*		
PDE (2)	Harsh touch, texture	http://wormatlas.org/neurons/Individual%20Neurons/PDEframeset.html
*Posterior body (not associated with sensilla)*		
PHC (2)	Noxious heat	http://wormatlas.org/neurons/Individual%20Neurons/PHCframeset.html
PLM (2)	Gentle touch	http://wormatlas.org/neurons/Individual%20Neurons/PLMframeset.html
PVM (1)	Gentle touch	http://wormatlas.org/neurons/Individual%20Neurons/PVMframeset.html
PQR (1)	Harsh touch	http://wormatlas.org/neurons/Individual%20Neurons/PQRframeset.html
*Innervation of both anterior and posterior body (not associated with sensilla)*		
ALA (1)	Harsh touch	http://wormatlas.org/neurons/Individual%20Neurons/ALAframeset.html
PVD (2)	Harsh touch, noxious cold	http://wormatlas.org/neurons/Individual%20Neurons/PVDframeset.html

a Chemosensory properties of a subset of neurons in this Table are discussed in a forthcoming WormBook chapter.

## Mechanosensation and Sensory Mechanotransduction

All animals are endowed with sensory neurons specialized to detect mechanical energy in the form of touch, potential injury (nociception), or body movement (proprioception). The proper function of internal organs also depends on feedback from mechanoreceptor neurons (interoreception). Mechanoreceptor neurons differ in their anatomy and intimate association with skin, muscle, and internal organs, but share the vital function of performing mechanotransduction. Studies in *C. elegans* were the first to use unbiased, forward genetic screens to identify proteins specifically required for mechanosensation (Chalfie and Sulston 1981; Chalfie and Au 1989) and the first to combine genetic dissection with optical imaging (Suzuki *et al.* 2003) and electrophysiology (O’Hagan *et al.* 2005) of identified mechanoreceptor neurons in living animals. Readers interested in how mechanosensory transduction in *C. elegans* relates to parallel processes in other animals are referred to other sources (Arnadóttir and Chalfie 2010; Katta *et al.* 2015; Wu *et al.* 2017).

**Figure 2:**
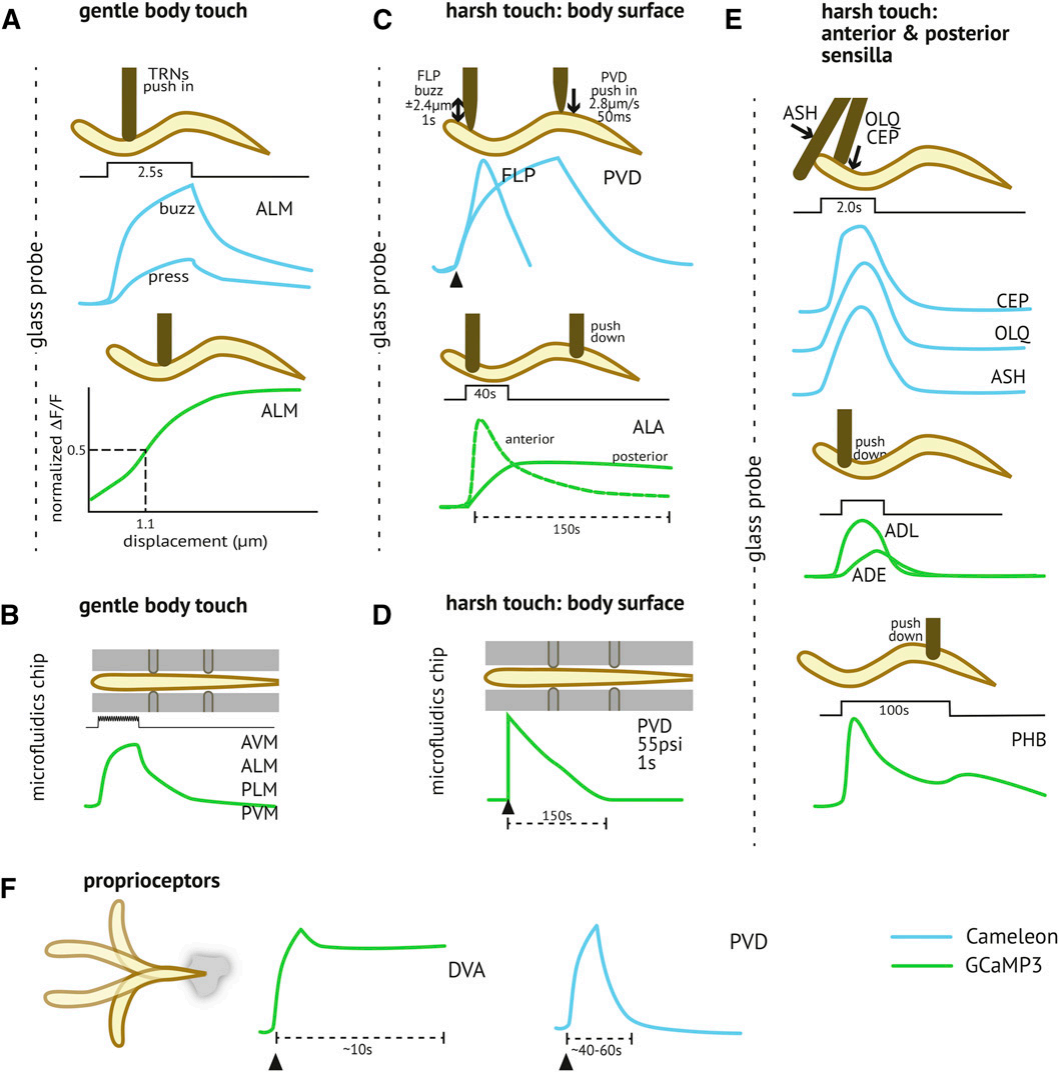
Mechanical stimuli activate calcium transients in mechanoreceptor neurons. Schematics depict the stimulation position, intensity, and dynamics as well as calcium transients monitored using the ratiometric indicator, Cameleon (blue), or the single-wavelength indicator GCaMP (green). (A) Touch receptor neurons respond to a simple stimulus (press) and more strongly to a complex one (buzz) delivered via glass probe. Based on calcium imaging, the ALM neurons can detect submicrometer displacements (bottom). Data source(s): (Suzuki *et al.* 2003; Chatzigeorgiou *et al.* 2010; Chen and Chalfie 2014). (B) Touch receptor neurons activated in a microfluidic chip also demonstrate stronger response to buzz stimuli. Data source(s): (Cho *et al.* 2017; Nekimken *et al.* 2017a). (C) High intensity or harsh touch stimuli activate multidendritic and simple nociceptors. Each panel shows the time course of calcium transients evoked by mechanical stimuli delivered by pushing a stiff glass probe into the dorsal or ventral side of an immobilized animal (PVD, FLP) or pushing a probe down onto the side of an animal (ALA). Data source(s): (Chatzigeorgiou *et al.* 2010; Sanders *et al.* 2013; Cho *et al.* 2017). (D) Harsh touch stimuli delivered in a microfluidic chamber (PVD). Data source(s): (Cho *et al.* 2017), (E) High intensity or harsh touch stimuli activate nociceptors innervating anterior and posterior sensilla. Each panel shows the time course of calcium transients evoked by mechanical stimuli delivered by pushing a stiff glass probe into the dorsal or ventral side of an immobilized animal (ASH, OLQ, CEP) or by pushing down in an anterior (ADE, ADL) or posterior position (PHB) Data source(s): (Kindt *et al.* 2007a,b; Chatzigeorgiou *et al.* 2010; Chatzigeorgiou and Schafer 2011; Sanders *et al.* 2013; Zou *et al.* 2017). (F) Proprioceptors activated during body bending. Data source(s): (Li *et al.* 2006; Albeg *et al.* 2011).

**Figure 3:**
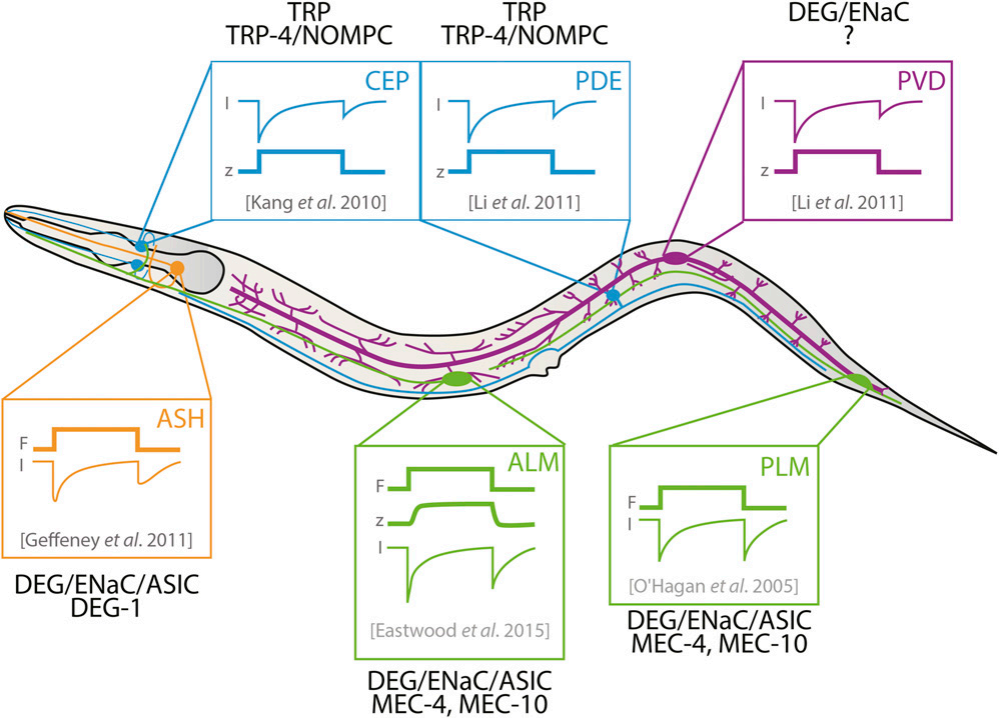
Dynamics of mechanoreceptor currents (MRCs) recorded from *C. elegans* neurons *in vivo*. Shown (schematically) are the first reported measurements of MRCs in PDE (Li *et al.* 2011), ASH (Geffeney *et al.* 2011), ALM (Eastwood *et al.* 2015), CEP (Kang *et al.* 2010), PLM (O’Hagan *et al.* 2005), and PVD (Li *et al.* 2011). MRCs in ADE and CEP depend on expression of the *trp-4* gene, which encodes the *C. elegans* homolog of the *Drosophila* NOMPC mechanosensitive ion channel. MRCs in ASH, ALM, PVD, and PLM are all carried by sodium ions and blocked by the diuretic drug, amiloride, and depend on DEG-1 in ASH and on MEC-4 in ALM and PLM. [The *mec-10* gene is dispensable for MRC generation in ALM and PLM, but contributes to the pore; the pore forming subunits of MRCs in PVD remain to be discovered].

*C. elegans* hermaphrodites have 45, and males have an additional 42, putative mechanoreceptor neurons (Figure 1 and Table 1). Responses to mechanical stimulation have been observed using calcium imaging in ca. one-third of these sensory neurons (see Figure 2 and text below). Each neuron is embedded in a specific place in the body and specialized to detect mechanical stresses that originate in that location. They differ in their sensitivity to mechanical loads: some detect low-intensity, gentle touch, while others detect high-intensity, harsh stimuli. As in other animals, *C. elegans* mechanoreceptors are believed to rely on ion channels gated by mechanical cues. Techniques for patch-clamp recordings from identified *C. elegans* neurons (Goodman *et al.* 1998) have been adapted to measure mechanoreceptor currents (MRCs) in several mechanoreceptor neurons (Figure 3).

The integration of mechanosensory neurons into neural circuits, and how behavioral responses are linked to external and self-generated mechanical cues are described elsewhere (*e.g.*, Goodman 2006; Schafer 2015). In reviewing current knowledge, we also seek to alert readers to gaps in knowledge and to unifying concepts that are emerging from recent research. For instance, current evidence suggests that ion channels are the principal receptors for light touch, painful or harsh touch, and proprioception, and that the proximal effect leading to their activation is indentation-induced changes in local mechanical strain (*aka* stretch).

### Gentle or light touch

The gentle touch receptor neurons (TRNs) innervate the body surface and are required for touch-evoked avoidance behaviors. Optogenetic activation of these neurons is sufficient to evoke avoidance behaviors (Leifer *et al.* 2011; Stirman *et al.* 2011). Activation of the anterior TRNs suppresses rapid head swings during backward movement, an effect thought to enable nematodes to escape from traps set by predatory fungi (Maguire *et al.* 2011). The TRNs also contribute to habituation (reviewed in Bozorgmehr *et al.* 2013), a form of behavioral plasticity in which animals become less sensitive to repeated sensory stimulation. The simplicity of TRN-dependent behaviors enabled Chalfie and colleagues to perform comprehensive, forward genetic screens that identified hundreds of mutant alleles that impair or eliminate touch sensitivity in *C. elegans* hermaphrodites (Chalfie and Sulston 1981; Chalfie and Au 1989). In turn, these so-called *mec* (*mec*hanosensory abnormal) mutants have been critical tools for advancing our understanding of mechanical senses more generally.

Freely moving wild-type animals can respond to forces as small as 100 nN and the response probability saturates near 0.8 (or 8 of 10 trials) for forces exceeding 2 µN (Petzold *et al.* 2013). The force required for half-activation (*F*_1/2_), depends on body stiffness, indicating that a complex relationship exists between applied force and touch-evoked behavior. Indeed, touch sensitivity is more directly related to body indentation than it is to applied force. An indentation of only 100 nm is sufficient to evoke an avoidance response and ∼450 nm is the indentation required to evoke a half-maximal response (Petzold *et al.* 2013). Thus, *C. elegans* hermaphrodites are extraordinarily sensitive to mechanical stimuli and touch sensation depends on skin indentation, rather than the applied force.

#### TRN cytoskeleton and extracellular matrix:

Wild-type TRN neurites contain a cross-linked bundle of ca. 40–50 microtubules; individual microtubules are long (10–15 µm) and staggered to fill the length of the neurite (Chalfie and Thomson 1979). TRN microtubules are composed of 15 protofilaments and are highly acetylated (Chalfie and Thomson 1982; Fukushige *et al.* 1999). The *C. elegans* genome contains two genes encoding α-tubulin acetyltransferases, *mec-17* and *atat-2*, and both paralogs are coexpressed in the TRNs (Akella *et al.* 2010; Shida *et al.* 2010; Topalidou *et al.* 2012). The TRNs express multiple α- and β-tubulin isoforms, including four α-tubulins, *tba-1*, *tba-2*, *tba-7*, *mec-12*, and three β-tubulins, *tbb-1*, *tbb-2*, *mec-7* (Savage *et al.* 1989; Fukushige *et al.* 1995, 1999; Lockhead *et al.* 2016; Zheng *et al.* 2017). The *mec-12* α-tubulin and *mec-7* β-tubulin genes are expressed at levels more than fivefold higher than other tubulin genes in TRNs (Lockhead *et al.* 2016), underscoring their critical importance in TRN development and function. Loss of *mec-12* or *mec-7* function is sufficient to disrupt 15-protofilament microtubules in the TRNs (Chalfie and Thomson 1982; Chalfie *et al.* 1986; Cueva *et al.* 2012; Zheng *et al.* 2017). Null mutants of both genes are touch-insensitive or Mec (Chalfie and Au 1989) and have mechanoreceptor currents that are dramatically reduced in amplitude (O’Hagan *et al.* 2005; Bounoutas *et al.* 2009). These tubulins also play critical roles in vesicle transport, as demonstrated by transport defects in animals carrying missense mutations in both *mec-7* and *mec-12* (Krieg *et al.* 2017; Zheng *et al.* 2017). The contribution of other tubulins to TRN development and function may be partially redundant or specialized. *tba-1;mec-12* double mutants exhibit stronger defects in axon outgrowth than either single mutant (Lockhead *et al.* 2016), and *tba-7* mutants exhibit ectopic neurites (Zheng *et al.* 2017).

In newly hatched larvae, the lateral TRNs (ALM, PLM) lie next to muscle cells. As animals mature and epidermal cells expand, TRN neurites separate from the muscle. This process is disrupted in *him-4* hemicentin mutants (Vogel and Hedgecock 2001) and in *mec-1* mutants with defects in the N-terminal portion of the encoded MEC-1 protein (Emtage *et al.* 2004). The *mec-1* gene produces several transcripts and the longer ones encode a large polypeptide of >2000 amino acids. Consistent with its influence on neuronal attachment and the presence of a predicted N-terminal signal sequence, MEC-1 is thought to reside in the ECM. Indeed, *mec-1* mutants lack the electron-dense mantle (Chalfie and Sulston 1981). Two additional proteins are thought to contribute to the specialized ECM: MEC-5 and MEC-9. The MEC-5 protein is an atypical collagen that is not expressed by the TRNs, but is required for the proper distribution of MEC-4 channels (see below) along the TRNs (Du *et al.* 1996; Emtage *et al.* 2004) and for touch-evoked mechanoreceptor currents (O’Hagan 2005). A long isoform of the MEC-9 protein harbors multiple Kunitz and EGF domains and is expressed selectively in the TRNs (Du *et al.* 1996). As found for MEC-5, loss of MEC-9 also disrupts the distribution of MEC-4 channels and the production of touch-evoked mechanoreceptor currents (Emtage *et al.* 2004; O’Hagan 2005).

#### TRN physiology—touch-evoked calcium signals:

One of the earliest applications of genetically encoded calcium indicators in living animals involved expressing Cameleon, a FRET-based calcium indicator (Miyawaki *et al.* 1997) in *C. elegans* muscles (Kerr *et al.* 2000) and in the TRNs (Suzuki *et al.* 2003). By imaging touch-evoked changes in Cameleon signals, Suzuki *et al.* (2003) showed that mechanical stimulation activated the TRNs (Figure 2A, top). Touch-evoked calcium signals were abolished by null mutations in the *mec-4*, *mec-2*, and *mec-6* genes previously known to abrogate touch sensation, and impaired by reduction-of-function mutations in the *egl-19* voltage-gated calcium channel (Suzuki *et al.* 2003). This study was the first to establish that any of the putative mechanosensory neurons in *C. elegans* function as *bona fide* mechanoreceptor neurons, and to link genes encoding the ion channel proteins, *mec-4*, *mec-2*, and *mec-6* to touch-evoked activation of the TRNs. As illustrated schematically in Figure 2A (top), this study also showed that a brief, sinusoidal stimulus (buzz) was more effective than a single pulse (press).

The amplitude of touch-evoked calcium transients decreases during repeated stimulation; this effect is stronger in ALM than it is in PLM (Kindt *et al.* 2007a). Subsequent studies using more sensitive genetically encoded calcium indicators (*e.g.*, GCaMP3, GCaMP6s) showed that calcium transients increase with probe displacement (Figure 2A, bottom) and that response amplitude, but not displacement sensitivity, is decreased by mutations in the EGL-19 L-type voltage-gated calcium channel (Chen and Chalfie 2014). The latter finding is consistent with the idea that voltage-gated calcium channels amplify touch-evoked calcium transients, but do not determine mechanosensitivity.

#### TRN physiology:

Touch depolarizes the ALM and PLM neurons and activates an inward mechanoreceptor current (MRC) at both the onset and withdrawal of mechanical loads (O’Hagan *et al.* 2005; Bounoutas *et al.* 2009; Arnadottir *et al.* 2011; Chen and Chalfie 2015; Eastwood *et al.* 2015; Chen *et al.* 2016a; Han *et al.* 2017) (Figure 3). These response dynamics are not unique to the TRNs; rather they are shared by other mechanoreceptor neurons (Figure 3). Given that a buzz or brief sinusoidal stimulus activates MRCs about twice during each cycle (Eastwood *et al.* 2015), these response dynamics are likely to account for the empirical observation that a buzz generates larger calcium signals than a press (Suzuki *et al.* 2003; Nekimken *et al.* 2017a). Studies using a feedback-control device to control applied force and measure indentation (or vice versa) show that MRC activation depends on the indentation produced, rather than the force applied (Petzold *et al.* 2013; Eastwood *et al.* 2015). Like the responses of mammalian Pacinian corpuscles (Sato 1961; Loewenstein and Mendelson 1965), MRC activation is also velocity- and frequency-dependent: neither pushing slowly against the animal’s body nor a brief, sinusoidal stimulus of <3 Hz could activate any current. Thus, *C. elegans* TRNs appear to be tuned to fast stimulation and are insensitive to slow stimuli such as those generated during movement, and, therefore, unlikely to function as proprioceptors. Consistent with this prediction, animals that lack TRNs have no obvious defects in movement.

In the TRNs, mechanoreceptor currents depend on extracellular sodium ions and are inhibited by the diuretic drug, amiloride. These properties are shared by channels formed by members of the superfamily of DEG/ENaC/ASIC channel proteins (Eastwood and Goodman 2012; Kellenberger and Schild 2015; Boscardin *et al.* 2016), including MEC-4 (Goodman *et al.* 2002). Loss of MEC-4 eliminates MRCs (O’Hagan *et al.* 2005), while loss of MEC-10 has only minor effects on these touch-evoked currents (Arnadottir *et al.* 2011). Mutations affecting conserved glycines in the second transmembrane domain of MEC-4 and MEC-10 alter the ionic permeability of MRCs recorded in the TRNs (O’Hagan 2005; Arnadottir *et al.* 2011), demonstrating that these proteins are pore-forming subunits of the native mechano-electrical transduction (MeT) channel responsible for touch sensation in the TRNs. This study was the first to link specific ion channel subunits to a native MeT channel in any mechanoreceptor neuron.

### Harsh touch (mechanical nociception)

Harsh touch is detected primarily by sensory neurons innervating the nose and by multidendritic neurons that tile the body surface (Figure 1 and Table 1), and is linked most often to avoidance behaviors. For instance, head-on collisions evoke a robust evasive behavior consisting of a quick reversal followed by an omega turn, a behavior commonly described as a nose-touch response. Laser-mediated ablation studies reveal that this response depends on the ASH, CEP, and FLP neurons (Kaplan and Horvitz 1993). Optogenetic activation of the ASH neurons alone (Guo *et al.* 2009) or the FLP neurons alone (Li *et al.* 2011) is sufficient to evoke a reversal response. Mechanical stimuli delivered along the ventral side of the animal’s nose evoke head withdrawal and depend on the OLQ, FLP, and IL1 neurons (Hart *et al.* 1995). The OLQ, FLP, and CEP neurons are interconnected through gap junctions with RIH, a so-called hub interneuron, and this network appears to operate in parallel with the ASH neurons to govern sensorimotor integration in a complex manner (Chatzigeorgiou and Schafer 2011; Rabinowitch *et al.* 2013).

FLP and PVD are bilaterally symmetric, multidentric sensory neurons that extend primary dendrites bearing menorah-shaped dendritic structures that extend laterally across the body surface and are embedded between body wall muscles and the epidermal layer (Figure 1) (Halevi *et al.* 2002; Albeg *et al.* 2011). In contrast with FLP, optogenetic activation of PVD triggers forward movement (Li *et al.* 2011). The PVD and FLP neurons mediate responses to high-intensity mechanical cues (harsh touch) as well as to extreme cold (PVD) and heat (FLP) (Way and Chalfie 1989; Chatzigeorgiou *et al.* 2010; Albeg *et al.* 2011; Chatzigeorgiou and Schafer 2011; Schild *et al.* 2014).

Based on laser killing experiments (Li *et al.* 2011), these other sensory neurons have been implicated in harsh touch avoidance behaviors: ADE, AQR, BDU, SDQ, PHA, PHB, PQR, and PDE. It is not yet clear how mechanical loads affect signaling by these neurons or whether they function as primary mechanoreceptors.

#### Cellular responses to harsh touch—calcium signals:

Compressing the worm’s nose or body evokes transient increases in intracellular calcium in certain neurons and some glia linked to harsh touch sensation. The ASH neurons are activated by rapid compression delivered perpendicular to the anterior-posterior body axis (Kindt *et al.* 2002; Hilliard *et al.* 2005; Walker *et al.* 2009), which is illustrated schematically in Figure 2E. Their response is independent of *unc-13*-dependent synaptic transmission (Hilliard *et al.* 2005) and decreased in *itr-1* IP3 receptor mutants (Walker *et al.* 2009). These findings imply that ASH is a primary mechanoreceptor neuron and that release of calcium from ITR-1-dependent intracellular stores contributes to compression-evoked calcium transients. Mechanical stimuli similar to those that activate the ASH neurons also evoke large inward currents and calcium transients in the amphid sheath cells (Ding *et al.* 2015). These observations raise the possibility that ASH signals downstream of glia or that ASH mechanoresponses involve signals from both non-neuronal cells (glia) and sensory neurons. A scenario analogous to the second possibility occurs in mammals in which both specialized epidermal cells and the neurons that innervate them function as primary mechanoreceptors (Reviewed by Vásquez *et al.* 2014; Woo *et al.* 2015).

The OLQ (Kindt *et al.* 2002; Chatzigeorgiou and Schafer 2011) and CEP neurons (Kindt *et al.* 2007b) also behave like primary mechanoreceptor neurons (Figure 2E and Table 1). Mechanoresponses in OLQ and CEP depend on the *osm-9* TRPV channel and the *trp-4* TRPN channel, respectively (Chatzigeorgiou and Schafer 2011). Consistent with the idea that these channels are needed for mechanosensitivity, OLQ mechanoresponses are restored by expression of *osm-9* in the OLQ neurons and CEP mechanoresponses are restored by expression of *trp-4* in the CEP neurons (Chatzigeorgiou and Schafer 2011). Compressing the nose in both a harsh and gentle manner activates calcium transients in FLP (Chatzigeorgiou and Schafer 2011) (Figure 2C). Sensitivity to modest or gentle indentation is facilitated by two other mechanoreceptor neurons, OLQ and CEP, via the common hub neuron, RIH (Chatzigeorgiou and Schafer 2011). By contrast, sensitivity to harsh stimuli is thought to be cell autonomous based on a requirement for the expression of an intact *mec-10* DEG/ENaC/ASIC channel gene in FLP itself (Chatzigeorgiou and Schafer 2011).

Body indentation evokes calcium transients in PVD (Chatzigeorgiou and Schafer 2011) whose amplitude increase with stimulus intensity and stimulus duration (Cho *et al.* 2017) (Figure 2C). However, PVD neurons are also activated by body bends (Albeg *et al.* 2011) (Figure 2F)—a result that suggests that these neurons may also function as proprioceptors. Consistent with this idea, genetic ablation of the PVD neurons alters body posture (Albeg *et al.* 2011) and affects locomotion speed (Cohen *et al.* 2012). It will be interesting to discover if both classes of multidendritic neurons, PVD and FLP, have dual roles as nociceptors and proprioceptors, and how the behaviors linked to activation of these mechanoreceptor neurons depend on neural circuitry. The shape of the ALA neuron is much simpler than that of PVD and FLP (Figure 1), but it has one of the longest neurites in the *C. elegans* nervous system. Calcium imaging (Figure 2C) demonstrates that ALA neurites are sensitive to high-intensity (harsh touch) mechanical stimulation along their lengths, and that distal stimuli generate slower responses than proximal stimuli (Sanders *et al.* 2013). As shown schematically in Figure 2E, mechanical stimuli evoke calcium transients in two other classes of sensory neurons not previously considered mechanoreceptors: PHA/PHB, ADL (Sanders *et al.* 2013).

#### Cellular responses to harsh touch—mechanoreceptor currents:

The finding that compressing the worm’s nose evokes transient increases in intracellular calcium implies that mechanical stimuli depolarize mechanoreceptor neurons by activating inward currents. Indeed, the onset and withdrawal of such mechanical loads activates an inward mechanoreceptor current (MRC) in the CEP neurons (Kang *et al.* 2010; Han *et al.* 2017) (Figure 3). The channels carrying these currents are permeable to both Na^+^ and K^+^ ions and depend on expression of the *trp-4* gene encoding the *C. elegans* ortholog of the mechanosensitive NOMPC protein (Kang *et al.* 2010). MRC activation is displacement-dependent and adapts to a conditioning stimulus by shifting its activation curve to larger displacements (Kang *et al.* 2010). Polymorphisms in the *trp-4* gene may govern the relationship between MRC activation and displacement (Han *et al.* 2017).

As shown schematically in Figure 3, electrophysiological recordings from ASH reveal robust mechanoreceptor potentials and sodium-dependent, amiloride-sensitive mechanoreceptor currents (Geffeney *et al.* 2011; Ding *et al.* 2015). As found in the touch receptor neurons (O’Hagan *et al.* 2005) and CEP neurons (Kang *et al.* 2010), MRCs activate with a millisecond latency and occur in response to both the onset and removal of mechanical deformations of the nose (Geffeney *et al.* 2011). They are dramatically reduced in mutants carrying a deletion in the *deg-1* DEG/ENaC/ASIC gene. Unexpectedly, MRCs are unaffected by loss of the *osm-9* or the *ocr-2* TRPV channel genes alone or in combination (Geffeney *et al.* 2011). This counterintuitive result implies that these two TRPV channels contribute to ASH signaling downstream of the initial transduction event. Similar to the TRNs and ASH, electrical recordings from PVD and PDE demonstrate that mechanical stimulation activates an inward current at both the onset and removal of the stimulus. Mechanoreceptor currents in these cells are blocked by amiloride and likely to be carried primarily by sodium ions (Li *et al.* 2011).

Although electrophysiological recordings have not been reported for any other sensory neuron linked to harsh touch sensation in *C. elegans*, recordings from the amphid sheath cells reveal large, mechanically activated inward currents. These currents reverse polarity near 0 mV and are not sensitive to amiloride (Ding *et al.* 2015)—properties that make it unlikely that they are carried by a DEG/ENaC/ASIC channel. The protein partners that make up this channel are not currently known, nor is it known how mechanosensitivity in these cells contributes to harsh touch sensation in particular or to other functions more broadly.

### Texture sensing

Wild-type animals can discriminate between different surface textures in a manner that depends on CEP and ADE in the nose and PDE in the tail (Sawin *et al.* 2000; Han *et al.* 2017). As noted above, CEP and PDE generate currents in response to both the onset and withdrawal of an indentation (Kang *et al.* 2010; Li *et al.* 2011). This response pattern is consistent with mechanoreceptor neurons being sensitive to the temporal dynamics of mechanical stimulation. In this context, it is tempting to speculate it is these response dynamics that enable CEP, ADE and PDE to detect the vibration-like mechanical stimuli produced as the worm crawls over a textured surface.

### Proprioception

The role of proprioception in regulating gait in *C. elegans* has been investigated using both experimental and computational approaches (reviewed by Schafer 2015; Zhen and Samuel 2015). One model emerging from this effort is that the B class of motor neurons helps to propagate signals related to body curvature (Wen *et al.* 2012), but it is not known if their activity is directly affected by the mechanical stresses generated during movement. The DVA (Kang *et al.* 2010) and PVD (Albeg *et al.* 2011; Cohen *et al.* 2012) neurons have also been linked to proprioception (Figure 2F). In addition to these neurons innervating the central and posterior domains of the worm’s body, the fourfold symmetric SMD neurons innervating the head were also proposed to function as proprioceptors based upon their position in the body and neural network (White *et al.* 1986). Consistent with such a function, the SMDD and SMDV neurons are activated by dorsal and ventral bending, respectively, and are activated in an antiphase manner during forward locomotion (Yeon *et al.* 2018). The sensitivity of the SMDD neurons to body bending, and the coordination of the SMDV and SMDD neurons, depend upon expression of TRP-1 and TRP-2 TRPC channels in the SMD neurons. Expression of TRP-1 confers sensitivity to dorsal-ventral bending on the AWC chemosensory neurons. Together, these findings suggest that a TRPC ion channel may function as a proprioceptive receptor in *C. elegans* neurons (Yeon *et al.* 2018).

### Mating and mechanoreceptor neurons

*C. elegans* males have 42 sex-specific, ciliated mechanoreceptor neurons that innervate the tail, hook, post-cloacal sensilla and spicules (Figure 1). Based on laser ablation studies (Liu and Sternberg 1995), all of these sensory neurons are thought to play essential roles in mating behavior. The sensilla that innervate the ventral surface of the male tail are required for responses to ventral contact with hermaphrodites, while dorsally directed rays mediate responses to dorsal contact with hermaphrodites (Liu and Sternberg 1995). The hook sensilla function in vulva location, while the sensory neurons that innervate the spicule are likely to provide feedback for spicule insertion into the vulva and subsequent sperm release. It is likely that many or perhaps all of these sensory neurons contribute to mate-sensing, while also providing proprioceptive feedback for ensuring the precise execution of mating behaviors, including response, turning, vulva detection, spicule insertion, and sperm transfer. For additional details regarding male mating behavior, please see Barr *et al.* (2018).

Like other mechanoreceptor neurons, the ray sensory neurons in the male tail respond to applied mechanical loads with a transient increase in intracellular calcium, as reported by GCaMP5 fluorescence in cell bodies (Zhang *et al.* 2018). Such responses are independent of the expression of *unc-13* and *unc-31* genes essential for release of clear- and dense-core synaptic vesicles, respectively (Zhang *et al.* 2018), indicating that touch-evoked calcium responses originate in the sensory neurons themselves. Calcium responses in the ray neurons are much slower than those of other *C. elegans* mechanoreceptors, reaching their peak amplitude over tens of seconds. Loss of the PKD-2 TRPP ion channel, which is required for male mating behavior (Barr *et al.* 2001), had no detectable effect on touch-evoked calcium signals. Loss of the OSM-9 TRPV channel decreased and slowed the mechanoreceptor calcium signals, but did not eliminate them. These signals were blocked by application of the DEG/ENaC/ASIC channel blocker, amiloride (Zhang *et al.* 2018). The picture emerging from these results is that, similar to ASH neurons in hermaphrodites, ray neurons in the male tail rely upon a DEG/ENaC/ASIC channel to detect mechanical stimuli and the OSM-9 TRPV channel to amplify such responses.

### Concluding remarks

These studies demonstrate that *C. elegans* relies on both TRP and DEG/ENaC channels to convert mechanical stimuli into electrical signals and that these channels activate in response to stimulus application and removal (summarized in Figure 3). Thus, divergent ion channels can function as mechanoelectrical transduction (MeT) channels (Katta *et al.* 2015). A corollary of this idea is that it is unlikely that all TRP or DEG/ENaC channels function as MeT channels. In support of this inference, the *osm-9* gene encoding a TRPV channel was initially thought to have this function (Colbert *et al.* 1997), but subsequent studies indicate that this TRPV channel is dispensable for mechanotransduction (Geffeney *et al.* 2011; Zhang *et al.* 2018). Future work will be needed to determine how this TRPV channel and others expressed in mechanosensory neurons contribute to mechanosensation.

Whether the *in vivo* activation of MeT channels depends on the force-from-lipid (Anishkin and Kung 2013; Anishkin *et al.* 2014), the force-from-filament (Katta *et al.* 2015), or a combination of both principles, remains to be determined. To date, none of the channels identified as sensory MeT channels in *C. elegans* have been shown to retain mechanosensitivity when purified and reconstituted. This is also true of most channels thought to function as MeT channels in mammals, with the exception of Piezo1 (Syeda *et al.* 2016). Even Piezo1 depends on an intimate relationship with the plasma membrane for mechanosensitivity (Guo and MacKinnon 2017; Haselwandter and MacKinnon 2018), suggesting that the mechanosensitivity of each MeT channel is a combined function of its biophysical properties and the cellular machinery. Owing to its diversity of mechanoreceptor neurons and the availability of techniques in single-neuron physiology and genetic dissection, studies in *C. elegans* are ideally suited to investigate whether a givent MeT channel is activated following the force-from-lipid or the force-from-filament principle or a combination of these biophysical mechanisms.

## Thermosensation and Molecular Mechanisms of Thermotransduction

Temperature is a ubiquitous stimulus that regulates the rate of every biochemical reaction. It is thus imperative for animals to sense temperature changes in order to appropriately alter their physiology and behavior. Thermosensation is particularly critical for ectotherms whose body temperature is dictated by the environment. Faced with large diurnal and seasonal temperature fluctuations, these animals must seek temperatures conducive to survival, and avoid noxious temperature ranges. Mechanisms underlying thermosensation are not fully understood. Members of the TRP family of cation channels have been implicated in thermosensation in both the physiological and noxious temperature ranges in multiple species, but species-specific thermosensory molecules and mechanisms have also been described (Sengupta and Garrity 2013; Li and Gong 2017; Hoffstaetter *et al.* 2018). The molecular basis of thermoreception in animals remains unknown.

*C. elegans* provides a particularly attractive system in which to study the molecular and neuronal basis of thermosensation. *C. elegans* is able to survive and reproduce across a relatively broad range of temperatures ranging from 12 to 26°, and can thus be considered a temperature generalist (Angilletta 2009; Anderson *et al.* 2011; Schulenburg and Felix 2017). These nematodes are remarkably thermosensitive and are able to detect, and behaviorally respond to, temperature changes of as little as 0.01° in the laboratory (Luo *et al.* 2006; Ramot *et al.* 2008a). In addition to regulating behavior, thermal cues regulate multiple aspects of nematode physiology. Studies in this organism have provided insights into the transduction pathways that allow animals to respond with high sensitivity and fidelity to temperature changes over a broad temperature range. Intriguingly, thermosensory pathways in *C. elegans* appear to share remarkable parallels with vertebrate phototransduction mechanisms, revealing a surprising conservation of signaling pathways that mediate responses to distinct sensory cues. This section reviews current knowledge about the neurons and signaling pathways that detect and translate environmental temperature changes into changes in locomotion, navigation, and physiology in *C. elegans*.

### Behavioral responses to thermal stimuli

**Figure 4:**
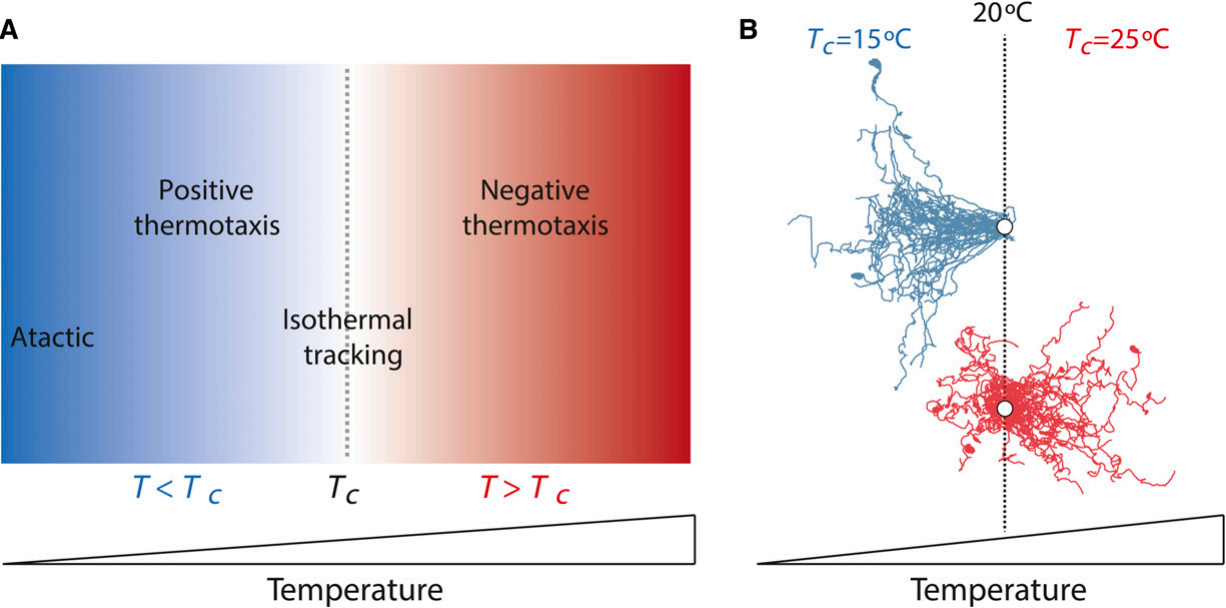
*C. elegans* exhibits *T_c_*-dependent navigation behaviors on spatial thermal gradients. (A) Schematic of navigation behaviors exhibited at temperatures relative to *T_c_* (*T_c_* is defined here as the temperature experienced 3–5 hr prior to the assay). *T*: ambient temperature. (B) Example trajectories of individual animals grown at either 15° (blue) or 25° (red) and placed at 20° on a shallow spatial thermal gradient. Note movement of animals toward cooler temperatures when *T > T_c_*, and movement toward warmer temperatures when *T < T_c_*. Trajectories are adjusted to the same starting point (white circles). Adapted from Luo *et al.* (2014a).

In the laboratory, the Bristol N2 strain of *C. elegans* does not exhibit a fixed preferred temperature. Instead, the behavior of worms on thermal spatial gradients is dictated by the animal’s previous temperature experience. When animals grown for several hours at a given temperature (cultivation temperature or *T_c_*) are placed at a temperature *T* > *T_c_*, worms migrate toward cooler temperatures on the gradient (negative thermotaxis) (Hedgecock and Russell 1975; Mori and Ohshima 1995). Conversely, when placed at a *T* that is a few degrees cooler than *T_c_* on shallow thermal gradients, they migrate toward warmer temperatures (positive thermotaxis) (Mori and Ohshima 1995; Ramot *et al.* 2008b; Jurado *et al.* 2010). However, if placed at *T* << *T_c_*, they are atactic (Ryu and Samuel 2002; Ramot *et al.* 2008b). In a narrow temperature band within 2° of *T_c_*, worms track isotherms (Hedgecock and Russell 1975; Mori and Ohshima 1995; Luo *et al.* 2006) (summarized in Figure 4). The exact behavior exhibited depends not only on the difference between *T* and *T_c_*, but also the gradient steepness and the animal’s internal state. Thus, on steep thermal gradients of >1.5°/cm, worms perform negative thermotaxis regardless of their temperature experience (Yamada and Ohshima 2003; Ramot *et al.* 2008b). In contrast, if animals are starved, while they continue to track isotherms, they no longer perform negative thermotaxis (Hedgecock and Russell 1975; Mohri *et al.* 2005; Biron *et al.* 2006; Chi *et al.* 2007). *T_c_* behavioral “memory” can be reset upon shifting adult animals to a new *T_c_* for a few hours (Hedgecock and Russell 1975; Mori and Ohshima 1995; Biron *et al.* 2006; Ramot *et al.* 2008b), indicating that this is a highly plastic process.

Detailed analyses of worm locomotion have described the navigation strategies underlying thermotaxis. Negative thermotaxis is mediated primarily via a biased random walk strategy (klinokinesis) (Ryu and Samuel 2002; Clark *et al.* 2007b; Ramot *et al.* 2008b; Luo *et al.* 2014a). In this strategy, worms extend the duration of their forward movement when moving toward cooler temperatures, and increase the frequency of reorientations when moving toward warmer temperatures (Ryu and Samuel 2002; Clark *et al.* 2007b). In addition, animals preferentially move toward cooler temperatures following a reorientation (Luo *et al.* 2014a). Movement toward warmer temperatures is mediated primarily via preferential movement toward warmer temperatures following reorientation; klinokinesis does not appear to contribute to this behavior (Luo *et al.* 2014a). Isothermal tracking is mediated via a distinct motor program. Worms do not seek isotherms but if they are serendipitously on an isotherm within *T*±∼2°, the temperature changes detected by the sinusoidal oscillation of their heads is translated via as yet uncharacterized mechanisms to suppress turns and maintain extended periods of forward movement (Ryu and Samuel 2002; Luo *et al.* 2006). Modeling experiments suggest that both negative thermotaxis and isothermal tracking behaviors are likely to contribute to the ability of worms to tolerate the daily temperature fluctuations they experience in their terrestrial habitats (Ramot *et al.* 2008b).

### Temperature responses in sensory neurons driving thermosensory navigation behaviors

Three pairs of sensory neurons (AFD, AWC, ASI) in the bilateral amphid sense organs of the head have been implicated in regulating thermosensory navigation behaviors (Table 1). Of these, the AFD neurons are the primary thermoreceptors regulating thermotaxis, with AWC and ASI playing minor modulatory roles.

#### Temperature responses in AFD:

**Figure 5:**
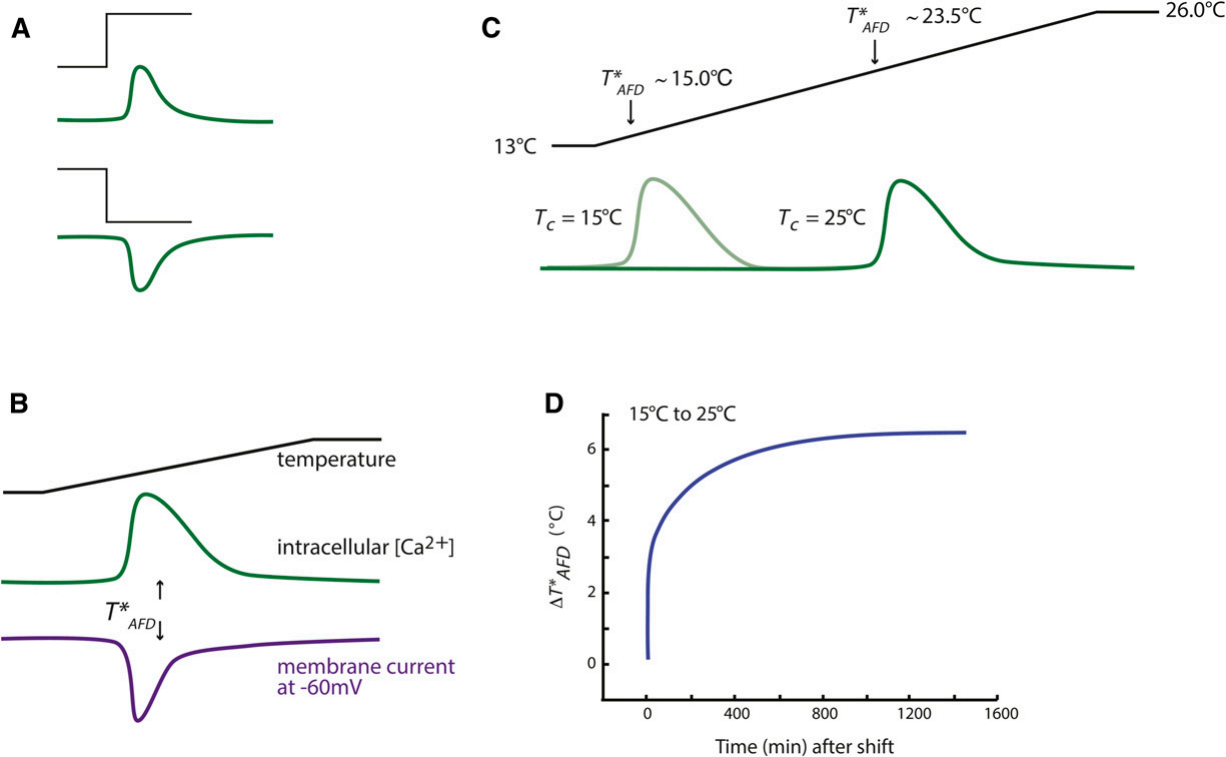
The AFD neurons exhibit responses to warming and cooling in a *T_c_* experience-dependent manner. (A) Schematic of changes in intracellular calcium in AFD (green lines) in response to a warming (top) or cooling (bottom) step (black lines) (Kimura *et al.* 2004; Clark *et al.* 2006). (B) Schematic of calcium transients (green) and thermoreceptor current (purple) (Ramot *et al.* 2008a) in response to a rising temperature ramp (black line). Responses are observed at temperatures above a *T_c_*-regulated threshold T*_AFD_. (C) T*_AFD_ shifts upon acclimation to a new *T_c_*. Schematic of calcium transients (green lines) in response to a rising temperature ramp (black line) in animals acclimated to different temperatures (Kimura *et al.* 2004; Biron *et al.* 2006; Clark *et al.* 2006). Adapted with permission from Goodman and Sengupta (2018). (D) Schematic showing dynamics of T*_AFD_ adaptation as measured by calcium transients upon temperature shift from 15 to 25°. Data source(s): (Yu *et al.* 2014).

Bilateral ablation of AFD fully abolishes isothermal tracking behavior and disrupts negative and positive thermotaxis, supporting a major role of this sensory neuron type in mediating thermotaxis (Mori and Ohshima 1995). Responses of AFD to temperature changes can be robustly measured using genetically encoded calcium indicators *in vivo*. Calcium levels in AFD rise as temperatures warm and fall when temperatures drop (Kimura *et al.* 2004; Clark *et al.* 2006) (Figure 5A). Temperature responses in AFD are strongly correlated with temperature fluctuations and can be observed in animals freely navigating a thermal gradient, as well as in immobilized animals subjected to temperature oscillations (Clark *et al.* 2006, 2007a; Tsukada *et al.* 2016; Venkatachalam *et al.* 2016). Moreover, these responses are observed in dissociated embryonic AFD neurons in culture (Kobayashi *et al.* 2016), indicating that these temperature responses are an intrinsic property of AFD.

Consistent with the observed calcium flux in AFD upon temperature fluctuations, warming and cooling increase and decrease a nonselective cation current, respectively (Figure 5B), resulting in depolarization of AFD upon warming, and hyperpolarization upon cooling (Ramot *et al.* 2008b). Quantification of the temperature dependence of the thermoreceptor current in AFD has shown that this neuron type is extraordinary in its thermosensitivity. The unitless value *Q_10_* (defined as the change in the rate of a reaction for every 10° change in temperature) provides a measure of the temperature-dependence of a reaction (Garrity *et al.* 2010). The *Q_10_* of warming-evoked thermoreceptor currents in AFD is >10^20^ (Ramot *et al.* 2008a). This value is reminiscent of those estimated for thermosensory neurons in pit viper snakes (Bullock and Diecke 1956) and is many orders of magnitude larger than that reported for mammalian thermosensory neurons (Vyklický *et al.* 1999) and individual thermosensitive TRP channels. This extraordinary thermal dependence implies the existence of a nonlinear amplification process that culminates in ion channel activation, similar to the signal cascade responsible for single-photon sensing in vertebrates.

Interestingly, similar to the *T_c_*-dependent modulation of the operating range of thermotaxis, the response threshold of AFD (T*_AFD_) is also *T_c_*-correlated (Kimura *et al.* 2004; Biron *et al.* 2006; Clark *et al.* 2006; Kobayashi *et al.* 2016). Thus, in animals grown at 15°, AFD responds to temperature changes when the temperature rises above ∼15°, whereas upon growth at 25°, T*_AFD_ shifts to ∼23° (Figure 5C). These response ranges of AFD correspond to the temperature ranges at which animals perform positive (*e.g.*, grown at 25°, and placed at *∼*22°) and negative (*e.g.*, grown at 15°, and placed at *T* > *T_c_*) thermotaxis behaviors (Luo *et al.* 2014b), raising the question of how similar thermosensory responses in AFD drive two distinct motor programs in different temperature ranges (see below). Neither T*_AFD_ nor temperature responses in AFD are altered upon starvation (Ramot *et al.* 2008a; Tsukada *et al.* 2016), indicating that feeding state disrupts negative thermotaxis behaviors downstream or in parallel to AFD thermosensory responses.

While resetting of behavioral *T_c_* memory to a new value requires growth at a new *T* for a few hours, T*_AFD_ can shift on two distinct timescales upon a *T* upshift (Figure 5D). Both calcium imaging and electrophysiology experiments have indicated that shifting animals to a higher *T* results in a very rapid (timescale of minutes) corresponding shift in T*_AFD_ (Ramot *et al.* 2008a; Yu *et al.* 2014; Hawk *et al.* 2018) (Figure 5D). If *T* >> *T_c_*, adaptation of T*_AFD_ to the final value additionally occurs on an hours-long timescale (Yu *et al.* 2014) (Figure 5D). In contrast to the biphasic T*_AFD_ adaptation upon *T* upshift, T*_AFD_ adapts slowly (timescale of hours) upon *T* downshift (Biron *et al.* 2006; Yu *et al.* 2014; Hawk *et al.* 2018). Rapid adaptation of T*_AFD_ upon *T* upshift may allow animals to retain the ability to respond to small temperature changes across a broad temperature range, whereas the slower adaptation rate may allow precise adaptation to the warmer *T_c_*.

While the motor program driving isothermal tracking behavior is unknown, temperature responses in AFD are also likely to be important for this behavior (Mori and Ohshima 1995). To track isotherms, animals must be able to detect and respond to rapid and small amplitude *T* changes around a constant *T* that is within ±2° of the *T_c_* (Luo *et al.* 2006). Indeed, AFD is able to reliably respond to sinusoidal temperature variations only near the *T_c_* (Wasserman *et al.* 2011). Moreover, the operating range for these responses is reset upon T*_AFD_ adaptation to a new *T* (Wasserman *et al.* 2011). These observations suggest that the ability of AFD to track small *T* changes around T*_AFD_ may drive isothermal tracking behavior.

#### Temperature responses in additional sensory neurons:

In addition to AFD, the AWC and ASI sensory neurons have been implicated in regulating thermosensory navigation behaviors (Biron *et al.* 2008; Kuhara *et al.* 2008; Beverly *et al.* 2011). Both these neuron types have been extensively studied in the context of mediating attraction to food-related odors (Bargmann and Horvitz 1991; Bargmann *et al.* 1993). Increased activity in AWC and ASI promotes and suppresses reorientations, respectively (Tsalik and Hobert 2003; Gray *et al.* 2005; Albrecht and Bargmann 2011; Larsch *et al.* 2013; Gordus *et al.* 2015). Consequently, physical or genetic ablation of AWC or ASI, or manipulation of their activity modulates, but does not fully disrupt, thermotaxis navigation primarily via alteration of reorientation frequency (Biron *et al.* 2008; Kuhara *et al.* 2008; Beverly *et al.* 2011). Consistently, AWC-ablated animals, or animals in which AWC is hyperactive, exhibit prolonged or abbreviated sojourns on isotherms, respectively (Biron *et al.* 2008). Both AWC and ASI respond to temperature changes, with the operating range of the responses dependent on *T_c_* (Biron *et al.* 2008; Kuhara *et al.* 2008; Beverly *et al.* 2011). However, unlike temperature responses in AFD, calcium dynamics in AWC or ASI are not phase-locked to non-nociceptive time-varying thermal stimuli (Biron *et al.* 2008; Kuhara *et al.* 2008; Beverly *et al.* 2011). Together, these results indicate that under defined conditions, AWC and ASI contribute to the modulation of thermotaxis behaviors.

### Molecular mechanisms of thermotransduction

Thermotransduction mechanisms have been studied more extensively in AFD than in AWC and ASI. It has long been established that cGMP signaling plays a critical role in thermotransduction based on the characterization of animals mutant for the *tax-2* and *tax-4* cGMP-gated channel subunit genes (Dusenbery *et al.* 1975; Hedgecock and Russell 1975). *tax-2* and *tax-4* mutants are atactic (Dusenbergy *et al*. 1975; Hedgecock and Russell 1975; Mori and Ohshima 1995), and the AFD neurons fail to exhibit changes in calcium dynamics (Kimura *et al.* 2004) or thermoreceptor current (Ramot *et al.* 2008a) in response to temperature changes in these mutants. Similarly, TAX-2 and TAX-4 are also essential for temperature responses in both AWC and ASI (Kuhara *et al.* 2008; Beverly *et al.* 2011).

TAX-2 and TAX-4 are unlikely to form temperature-gated channels. While these proteins are necessary for thermotransduction, they are expressed in multiple additional nonthermosensory neurons (Coburn *et al.* 1998; Kimura *et al.* 2004) indicating that they are not sufficient to mediate temperature responses. Moreover, a lag of ∼130 ms was observed between changes in temperature steps and opening of these channels in AFD (Ramot *et al.* 2008a), suggesting that these channels are gated by a soluble second messenger (such as cGMP) whose concentration is regulated in a temperature-dependent manner.

Soluble and transmembrane or receptor guanylyl cyclase (rGCs) enzymes catalyze the production of cGMP from GTP (Potter 2011; Kuhn 2016). Of the 27 rGCs encoded by the *C. elegans* genome, three (*gcy-8*, *gcy-18*, *gcy-23*) are expressed exclusively in AFD and are localized to their sensory endings (Yu *et al.* 1997; Inada *et al.* 2006; Ortiz *et al.* 2006). Similar to *tax-2* and *tax-4* mutants, animals mutant for all AFD-rGCs are atactic, and temperature changes fail to elicit changes in intracellular calcium or thermoreceptor current in the AFD neurons of these mutants (Inada *et al.* 2006; Ramot *et al.* 2008a; Wasserman *et al.* 2011; Wang *et al.* 2013; Takeishi *et al.* 2016). However, animals singly or doubly mutant for the AFD-rGCs exhibit subtle but nevertheless significant behavioral and imaging phenotypes (Inada *et al.* 2006; Wasserman *et al.* 2011; Wang *et al.* 2013; Takeishi *et al.* 2016). In particular, single and double mutant combinations of AFD-rGCs result in defects in thermotaxis navigation behaviors (Inada *et al.* 2006; Wasserman *et al.* 2011), and a lower T*_AFD_ (Wasserman *et al.* 2011; Takeishi *et al.* 2016). These observations suggest that AFD-rGCs act partly redundantly to mediate thermotransduction in AFD.

Are these AFD-rGCs themselves the thermosensors in AFD? Misexpression of either GCY-23 or GCY-18, but not GCY-8, was found to be sufficient to confer highly thermosensitive responses in chemosensory neurons and vulval muscles (Takeishi *et al.* 2016). Interestingly, the thresholds of response of GCY-23 and GCY-18 were distinct from each other upon misexpression in a chemosensory neuron type (Takeishi *et al.* 2016). Moreover, the response threshold of GCY-23 was cell type-specific and, unlike in AFD, largely uncorrelated with *T_c_* (Takeishi *et al.* 2016). Together, these results suggest that temperature may directly modulate AFD-rGC activity, but that both protein- and cell type-specific mechanisms define the temperature at which these proteins are activated. Whether other members of the rGC family similarly mediate thermosensation in AWC and ASI is not yet clear.

In addition to rGCs, several phosphodiesterase-encoding genes have been implicated in regulating thermotaxis behaviors. Of these *pde* phosphodiesterase genes, *pde-1*, *pde-2* and *pde-5* are expressed in AFD (Wang *et al.* 2013; Singhvi *et al.* 2016). In contrast to the lower T*_AFD_ of rGC mutants (Wasserman *et al.* 2011; Takeishi *et al.* 2016), T*_AFD_ in *pde-2* mutants is higher (Wang *et al.* 2013), similar to T*_AFD_ of animals grown on a nonhydrolyzable cGMP analog (Wasserman *et al.* 2011). Moreover, thermoreceptor currents in AFD are prolonged in *pde-2* mutants (Wang *et al.* 2013). These observations indicate that correct regulation of both rGC and PDE enzyme functions is essential for fine-tuning thermosensory properties of AFD. Whether similar to AFD-rGCs, temperature also modulates AFD-expressed PDE activity is not yet known.

**Figure 6:**
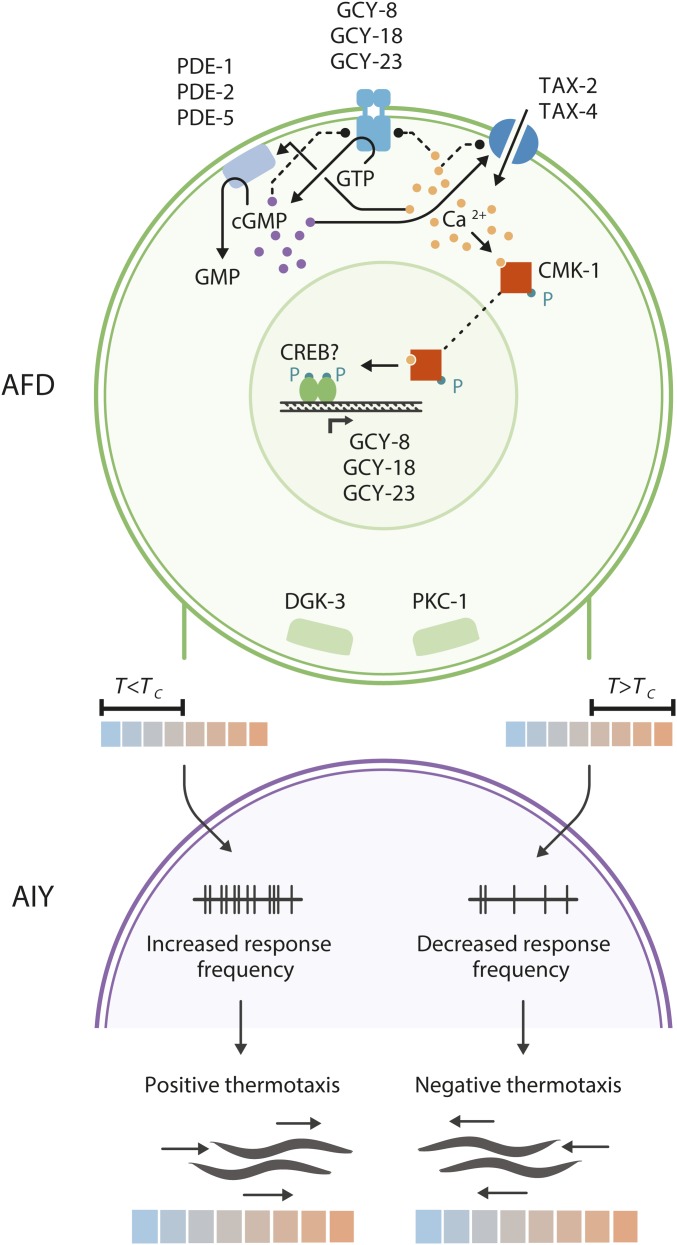
Schematic of thermosensory signal transduction and synaptic output of AFD. Upon warming, GCY-8, GCY-18, and GCY-23 are activated to increase intracellular cGMP levels. The TAX-2/TAX-4 cGMP-gated channels open and enable calcium ion influx and depolarization. cGMP is degraded by PDEs whose functions may also be temperature-dependent. Rapid adaptation is hypothesized to be mediated by cGMP and/or calcium-mediated feedback; targets of this feedback may be the rGCs, PDEs or the cGMP-gated channels. Long-term adaptation of the sensory response threshold is mediated in part via CMK-1-regulated changes in rGC and other gene expression. AFD synaptic output is also regulated upon long-term temperature acclimation via DGK-3 and PKC-1. The probability and amplitude of temperature-regulated responses in AIY, the major postsynaptic partner of AFD, is decreased at *T > T_c_* and promotes negative thermotaxis, whereas increased response probability in AIY at *T < T_c_* promotes positive thermotaxis. See text for additional details and references.

To summarize the current working model for thermotransduction in AFD, at T > T*_AFD_, thermosensory rGCs are activated to generate cGMP. Increased cGMP opens TAX-2/TAX-4 channels to permit cation influx and depolarization. Temperature changes and/or increased cGMP and/or calcium concentrations may also activate PDEs to hydrolyze cGMP and terminate signaling (summarized in Figure 6). The thermosensitivity of AFD-rGCs, amplification of a small temperature change via cGMP production, and rapid adaptation in part account for the extraordinary thermosensitivity of AFD. The molecular and cellular mechanisms of thermosensation in AFD are remarkably similar to those mediating light responses in vertebrate photoreceptors. Similar to AFD, photoreceptors are also highly sensitive; they can detect a single photon over a wide range of light intensities. It is tempting to speculate that these signaling pathways are an example of convergent evolution in cell types that are optimized for highly sensitive detection of environmental cues.

### Molecular mechanisms of thermosensory adaptation

As described above, T*_AFD_ is reset on both a short (minutes) and long (hours) timescale upon *T* upshift allowing these neurons to both retain responsiveness to small temperature changes across a broad temperature range, as well as to precisely shift their operating range in a *T_c_*-correlated manner. However, behavioral adaptation to *T_c_* is only observed upon a hours-long exposure to a higher *T*, suggesting that additional mechanisms downstream of T*_AFD_ adaptation—likely acting to modulate AFD synaptic output—must account for the altered behavioral operating range.

#### Mechanisms of adaptation of AFD thermosensory responses:

How does T*_AFD_ adapt on both a fast and slow timescale? The fast timescale of minutes precludes transcription-dependent mechanisms. Calcium buffering slows the timescale of this fast adaptation suggesting that calcium modulates this process (Ramot *et al.* 2008a). Consistent with this hypothesis, animals mutant for the calcium-regulated NCS-1 frequenin-like protein are defective in fast adaptation (Wang *et al.* 2013). Since frequenin modulates phosphodiesterase activity (Schaad *et al.* 1996), calcium-dependent modulation of AFD-expressed PDEs may reset T*_AFD_. In addition, manipulation of intracellular cGMP levels in AFD alters T*_AFD_ (Wasserman *et al.* 2011; Wang *et al.* 2013; Takeishi *et al.* 2016). Thus, a current notion is that upon temperature upshift, calcium and/or cGMP provide feedback signals to terminate signaling and rapidly reset T*_AFD_. Recent work has also implicated the SLO-1 BK^+^channels and the CNG-3 cGMP-gated channels in AFD adaptation (Aoki *et al.* 2018). The targets of this adaptation process are hypothesized to be the AFD-rGCs, AFD-expressed PDEs, cGMP-gated or K+ channels, or multiple combinations thereof (Figure 6).

Precise adaptation of T*_AFD_ upon a large T shift requires several hours and is mediated via changes in gene expression (Yu *et al.* 2014). Expression of AFD-rGCs is higher in animals grown at a warmer temperature, and this temperature-dependent change in gene expression requires the CMK-1 calcium/calmodulin-dependent protein kinase I gene (Yu *et al.* 2014) (Figure 6). In *cmk-1* mutants, expression of these (and likely additional genes) is not temperature-regulated, resulting in a decreased magnitude of T*_AFD_ adaptation and lower final T*_AFD_ (Yu *et al.* 2014). CMK-1-mediated changes in gene expression might require the Raf kinase pathway and possibly CREB (Nishida *et al.* 2011; Kobayashi *et al.* 2016) (Figure 6). Although T*_AFD_ adaptation has also been shown to occur in dissociated AFD neurons grown in culture (Kobayashi *et al.* 2016), cell nonautonomous mechanisms may also play a role in fine-tuning T*_AFD_ (see below).

#### Mechanisms of adaptation in AFD synaptic output:

The AIY interneurons are the primary postsynaptic partners of AFD (White *et al.* 1986). Thus, analyses of temperature responses in AIY can serve as a reasonable proxy for measurements of AFD synaptic output. The threshold of AIY temperature responses adapts on a behavioral hours-long timescale (Biron *et al.* 2006; Hawk *et al.* 2018) suggesting that adaptation of T*_AFD_ and AFD synaptic output can be partially decoupled molecularly. Indeed, mutations in the *dgk-3* diacylglycerol kinase and *pkc-1* nPKCε genes alter the rate of behavioral adaptation and AFD synaptic output without affecting T*_AFD_ adaptation (Okochi *et al.* 2005; Biron *et al.* 2006; Luo *et al.* 2014b; Hawk *et al.* 2018).

AFD to AIY synaptic transmission is modulated by the animal’s thermal experience in a complex manner. The AFD neurons are glutamatergic and are likely to signal to AIY via both glutamate-mediated inhibitory and peptidergic excitatory transmission (Clark *et al.* 2007b; Kuhara *et al.* 2011; Narayan *et al.* 2011; Ohnishi *et al.* 2011). Interestingly, in the operating range of negative thermotaxis at *T > T_c_*, both the probability and amplitude of AFD-driven AIY temperature responses are decreased, whereas conversely, in the operating range for positive thermotaxis, probability and amplitude of AIY responses are increased (Kuhara *et al.* 2011; Hawk *et al.* 2018) (Figure 6). The ranges in which these responses are exhibited in AIY adapt on a timescale corresponding to behavioral adaptation to *T_c_* (Hawk *et al.* 2018). How AFD-driven AIY responses are modulated bidirectionally as a function of *T_c_* experience is not yet clear.

### Nonautonomous regulation of AFD thermosensory properties

The complex sensory endings of AFD are comprised of multiple actin-based microvilli and a microtubule-based cilium; these endings are fully embedded in the processes of the amphid sheath cell at the worm nose (Perkins *et al.* 1986; Doroquez *et al.* 2014; Nguyen *et al.* 2014). The amphid sheath cells express the KCC-3 K^+^/Cl^−^ transporter which localizes around the AFD microvilli and regulates the concentration of extracellular Cl^−^ ions (Singhvi *et al.* 2016; Yoshida *et al.* 2016). Chloride ions in turn inhibit the basal activity of the GCY-8 AFD-rGC by directly binding its extracellular domain (Singhvi *et al.* 2016). Decreased GCY-8 activity and reduction of intracellular cGMP levels modulate actin cytoskeleton remodeling and elongate AFD microvilli (Singhvi *et al.* 2016). Conversely, in *kcc-3* mutants and upon constitutive activation of GCY-8, microvilli are shortened (Singhvi *et al.* 2016). While T*_AFD_ is unaffected in *kcc-3* mutants, AFD temperature response dynamics are markedly altered (Yoshida *et al.* 2016), and thermotaxis behaviors are disrupted (Singhvi *et al.* 2016; Yoshida *et al.* 2016). The amphid sheath cells have previously been shown to exhibit temperature-dependent, but AFD-independent, changes in gene expression (Procko *et al.* 2011). Thus, in one possible model, sheath cells could shape AFD temperature responses via temperature-dependent regulation of KCC-3 and extracellular Cl^−^ concentration. In addition to glia, AFD thermosensory properties may also be fine-tuned by temperature-regulated systemic signaling from the intestine (Nishida *et al.* 2011; Land and Rubin 2017).

### Temperature-dependent regulation of physiological processes

Environmental thermal stimuli regulate not only behavior but also physiological responses. These responses are mediated by AFD as well as additional thermosensory neurons. AFD has been implicated in coordinating systemic heat shock responses via serotonergic signaling (Prahlad *et al.* 2008; Tatum *et al.* 2015), and loss of AFD thermotransduction decreases thermotolerance (Prahlad *et al.* 2008). AFD also regulates temperature-dependent modulation of longevity. Warmer temperatures decrease the lifespan of ectotherms such as *C. elegans* (Lee and Kenyon 2009; Zhang *et al.* 2015). AFD antagonizes this heat-mediated shortening of lifespan via CMK-1-mediated upregulation of FLP-6 neuropeptidergic signaling in AFD at warmer temperatures (Lee and Kenyon 2009; Chen *et al.* 2016b). FLP-6 in turn increases lifespan via insulin and sterol hormone signaling (Chen *et al.* 2016b). Cold-dependent increase in lifespan is modulated in an AFD-independent manner and requires temperature sensation via the TRPA1 cation channel in the gut as well as in other as yet unidentified neurons (Xiao *et al.* 2013).

### Avoidance of noxious thermal stimuli

Similar to other animals, *C. elegans* avoid noxious cold or warm temperatures. Interestingly, avoidance is triggered not only by the absolute temperature but also by the rate of temperature change (Wittenburg and Baumeister 1999; Chatzigeorgiou *et al.* 2010; Glauser *et al.* 2011; Ghosh *et al.* 2012; Mohammadi *et al.* 2013; Schild and Glauser 2013). Thus, a rapid increase or decrease in temperature within or beyond the physiological temperature range can elicit avoidance (Chatzigeorgiou *et al.* 2010; Mohammadi *et al.* 2013). Detailed analyses of locomotion parameters have suggested that worms employ both avoidance and escape behaviors to decrease exposure to noxious heat (Schild and Glauser 2013).

Noxious heat avoidance has been analyzed using spatial or temporal thermal gradients or via the use of infrared lasers (Wittenburg and Baumeister 1999; Glauser *et al.* 2011; Mohammadi *et al.* 2013; Kotera *et al.* 2016). Multiple sensory neurons have been implicated in mediating noxious thermal avoidance (Table 1), possibly due to the use of diverse assays. AFD has been implicated in heat avoidance, and these neurons respond in a deterministic manner to stimuli that elicit avoidance (Liu *et al.* 2012; Kotera *et al.* 2016). In addition, the AWC neurons respond to noxious heat stimuli and are required for heat avoidance. Previous work has shown that the two AWC neurons are functionally asymmetric (referred to as AWC^ON^ and AWC^OFF^), and that this asymmetry is determined in a stochastic manner (Troemel *et al.* 1999; Wes and Bargmann 2001; Chalasani *et al.* 2007, 2016). Interestingly, these neurons also respond asymmetrically to oscillating thermal stimuli that elicit avoidance, such that only the AWC^OFF^ neuron is required for avoidance (Kotera *et al.* 2016). Additional neurons implicated in nociceptive thermal avoidance include the PVD (cold) (Chatzigeorgiou *et al.* 2010), FLP (heat) (Liu *et al.* 2012; Schild *et al.* 2014) and PHC (heat) (Liu *et al.* 2012) sensory neurons (Table 1). Of note, while the majority of these sensory neurons mediate avoidance of nociceptive thermal stimuli presented to the head of *C. elegans*, PVD and PHC have been suggested to mediate avoidance of thermal stimuli presented to the midbody and tail, respectively (Liu *et al.* 2012; Mohammadi *et al.* 2013). Unlike thermotaxis navigation behaviors, avoidance of nociceptive thermal stimuli requires TRP channels. TRPA1 (Chatzigeorgiou *et al.* 2010), as well as the TRPV channels OCR-2 and OSM-9 (Glauser *et al.* 2011; Liu *et al.* 2012), are required for avoidance of noxious temperatures, although it is not yet clear whether these channels are directly gated by temperature.

In parallel with the contribution of temperature experience in modulating thermosensory navigation behaviors, prior thermal history can also alter the range of noxious temperature responses. Acclimation at 28° for >1 hr shifts the threshold for warm avoidance to warmer values as compared to animals grown at 20° (Schild *et al.* 2014). Analogous to the role of CMK-1 CaMKI in mediating temperature experience-dependent alteration of the operating range of AFD (Yu *et al.* 2014), CMK-1 also acts in the FLP neurons to regulate the operating range of noxious heat avoidance (Schild *et al.* 2014). Similar to heat acclimation, acclimation to cooler temperatures promotes survival of *C. elegans* at low temperatures. Thus, while extended exposure to cold temperatures of 5° or lower kills *C. elegans*, growing animals at 15° for as little as 2–3 hr promotes survival (Ohta *et al.* 2014). Cold tolerance is negatively regulated by the bilateral ASJ sensory neuron pair in the head amphid organs (Ohta *et al.* 2014). ASJ responds tonically to a temperature change, with the amplitude of the response correlated with the acclimation temperature (Ohta *et al.* 2014). Temperature responses in ASJ are mediated by G proteins and the cGMP second messenger modulate insulin signaling to the intestine and other tissues to regulate cold tolerance (Ohta *et al.* 2014; Ujisawa *et al.* 2016).

### Concluding remarks

Analyses of thermosensation in *C. elegans* have identified rGCs as a new family of putative thermosensors. Intriguingly, the GC-G rGC has recently been implicated in sensing cooling in the mouse Grueneberg ganglion (Chao *et al.* 2015), suggesting that the functions of these molecules in sensing temperature may be conserved. It is currently unclear how the enzymatic activity of these molecules is regulated by temperature changes. How rapid adaptation of AFD temperature responses is mediated is also unknown. Given the conservation with vertebrate phototransduction mechanisms, it is possible that adaptation mechanisms similar to those operating in photoreceptors may also mediate temperature adaptation in AFD. Additional open questions include the molecular mechanisms driving long-term temperature adaptation of both the thermosensory and presynaptic thresholds of AFD, as well as the molecular components of thermotransduction pathways in neurons other than AFD. For additional details regarding AFD-mediated thermotransduction, we refer to the reader to other sources (Aoki and Mori 2015; Goodman and Sengupta 2018). Further studies of thermosensation in *C. elegans* are likely to reveal insights into both divergent mechanisms of thermotransduction, as well as the conservation between *C. elegans* thermosensory and vertebrate visual transduction pathways.

## Molecules and Neurons Mediating Responses to Magnetic and Electrical Fields, Light, and Humidity

### Sensing magnetic cues

Although many animals use the earth’s magnetic field to orient themselves and to travel long distances, molecular mechanisms of magnetoreception are remarkably poorly understood (Wiltschko and Wiltschko 2012; Mouritsen *et al.* 2016; Mouritsen 2018). Chains of magnetosomes containing magnetite have been shown to act as a compass and mediate magnetotaxis in magnetotactic bacteria (Blakemore 1975; Greene and Komeili 2012; Lefevre and Bazylinski 2013), but the identity and location of the magnetoreceptor in animals have been elusive and are a matter of ongoing debate. In *C. elegans*, the pair of AFD sensory neurons has been suggested to be magnetosensory (Vidal-Gadea *et al.* 2015) (Table 1), in addition to their well-described role in thermosensation. The AFD neurons respond weakly to earth-strength magnetic fields, and this response appears to be mediated via cGMP signaling (Vidal-Gadea *et al.* 2015). The three thermosensory rGCs GCY-8, GCY-18, and GCY-23, as well as the TAX-2 and TAX-4 cGMP-gated channels are required for the response of AFD to magnetic fields (Vidal-Gadea *et al.* 2015), suggesting that the signal is amplified and transduced via the cGMP second messenger. The identity of the magnetoreceptor in AFD is unknown, but both the cilia and microvilli located at the sensory endings of AFD are required for *C. elegans* to orient correctly in a magnetic field (Bainbridge *et al.* 2016). It has been posited that iron particles localized to the microvilli and/or in the surrounding glia may be directly associated with the membrane or transmembrane mechanosensory channels (Clites and Pierce 2017). Physical deformation of the membrane or channels induced by a magnetic field acting on the iron beads could then transduce an electrical signal (Clites and Pierce 2017). Although the ability of *C. elegans* to sense magnetic fields has been debated (Landler *et al.* 2018; Vidal-Gadea *et al.* 2018), it will be interesting to further corroborate this behavior and advance our knowledge of this poorly understood sensory modality in this genetically tractable organism.

### Sensing electrical cues

Electroreception has been extensively described in aquatic animals, but is less prevalent in terrestrial animals (Baker *et al.* 2013; Czech-Damal *et al.* 2013). *C. elegans* exhibits robust responses to imposed electrical fields (Sukul and Croll 1978; Gabel *et al.* 2007; Chrisman *et al.* 2016). These responses appear to be mediated by a distributed set of sensory neurons, with the specific neurons required dictated by the parameters of the imposed electrical field. Physical ablation of ASJ results in strong behavioral defects in electrosensory steering on a rotating electrical field (Gabel *et al.* 2007) (Table 1). Consistently, ASJ exhibits robust calcium responses in response to a rotating electrical stimuli (Gabel *et al.* 2007). Weak responses were also observed in ASH as well as in AWB, AWC, or ASK, although ablation of these neurons led to weak (ASH) or no (AWB, AWC, ASK) defects in electrosensory steering (Gabel *et al.* 2007). In contrast, electrotaxis on a fixed uniform electrical field appears to be mediated primarily by the functionally asymmetric AWC^ON^ member of the AWC sensory neuron pair, with a minor role for ASH (Chrisman *et al.* 2016). Electrosensory transduction pathways and their ethological function have yet to be uncovered.

### Sensing light

Although *C. elegans* lacks eyes, an obvious eyespot, or ocellus, it can detect and respond to light. For instance, ultraviolet light evokes a robust avoidance behavior that is similar to that observed following activation of mechanoreceptor neurons such as the TRNs, ASH, and PVD. UV light stimulation has also been proposed to modulate pharyngeal function by catalyzing the generation of reactive oxygen species (Bhatla and Horvitz 2015). Additionally, *C. elegans* may be sensitive to visible light—a recent study reported that *C. elegans* uses blue light to detect and avoid pathogenic bacteria based on their synthesis of colored compounds such as pyocyanin (Ghosh *et al.* 2017).

Remarkably, all of these light-dependent behaviors depend upon expression of the *lite-1* gene (Edwards *et al.* 2008; Liu *et al.* 2010; Ghosh *et al.* 2017). *lite-1* encodes a transmembrane protein homologous to insect gustatory receptors (Edwards *et al.* 2008; Liu *et al.* 2010). In the context of UV light mediated avoidance behavior, *lite-1* acts in the ASJ and ASH neurons and appears to depend on a G protein-mediated signal transduction pathway to exert its influence of these sensory neurons (Liu *et al.* 2010) (Table 1). The *lite-1* gene is also expressed at very high levels in the TRNs and linked to blue light-evoked increases in cytoplasmic calcium (Nekimken *et al.* 2017a). The LITE-1 protein is sufficient to confer UV sensitivity upon muscle cells (Edwards *et al.* 2008; Liu *et al.* 2010), suggesting that it functions as an autonomous photoreceptor. Consistent with this idea, purified LITE-1 protein efficiently absorbs light at 280 and 320 nm and missense mutations in the *lite-1* gene selectively affect absorption at 280 and 320 nm (Gong *et al.* 2016). Collectively, these findings indicate *C. elegans* and possibly other nematodes lacking eyes rely on sensory neurons to detect optical stimuli and use a membrane protein more closely related to insect gustatory receptors than to rhodopsin to detect photons.

### Humidity

Animals migrate toward the dry side on humidity gradients, even on gradients as shallow as 0.03% relative humidity per millimeter (Russell *et al.* 2014). This preference is somewhat plastic and is modulated by the relative humidity of their growth conditions as well as their satiety state (Russell *et al.* 2014). Although hygrosensation may be mediated via integration of mechanical cues due to changes in skin hydration, and temperature changes from evaporative cooling, ablation of either the mechanosensory FLP or thermosensory AFD neurons significantly impairs hygrotaxis in *C. elegans* (Russell *et al.* 2014) (Table 1). Consistently, hygrotaxis is impaired in animals mutant for molecules implicated in mechanosensation by FLP including MEC-10, ASIC-1, and MEC-6, as well as the TAX-4 cGMP-gated channel required for thermotransduction in AFD (Russell *et al.* 2014).

## Conclusions

Genetic dissection of sensory-guided behaviors and the responses of sensory neurons in *C. elegans* have provided detailed insights into the sensory signaling pathways that allow these nematodes to detect physical stimuli. *C. elegans* is highly sensitive to thermal and mechanical stimuli, while also being able to detect and respond to potential harmful (noxious) levels of these same stimuli. As in other animals, ion channels play key roles in sensory transduction; mechanical cues activate ion channels directly, while thermal fluctuations act indirectly through regulating the synthesis and degradation of soluble molecules (*e.g.*, cGMP). One theme that emerges from this work is that molecular pathways and signaling principles are remarkably conserved, although certain pathways can be deployed in different sensory contexts, and signaling principles may be preserved in the absence of conserved signal transduction pathways. For instance, the thermotransduction pathway used by AFD to detect thermal fluctuations of as little as <1° is remarkably similar to the one that enables vertebrate photoreceptors to detect single photons. Similarly, the response dynamics and frequency selectivity of the TRNs are reminiscent of those that govern the function of Pacinian corpuscles in mammals. The ASH, CEP, and PVD neurons share these properties, but rely on distinct genes to form ion channels activated by mechanical stimuli. Another theme that arises is that many of the sensory neurons in *C. elegans* are polymodal, responding to multiple types of physical and chemical stimuli (Table 1). The polymodal nature of these sensory neurons is perhaps unsurprising given that the animal accomplishes complex sensorimotor tasks with only 302 neurons. For instance, AFD responds to temperature, magnetic fields, and gases such as carbon dioxide (Bretscher *et al.* 2011), and is proposed to play a key role in humidity sensing (Russell *et al.* 2014) (Table 1). The ASH neurons are activated by noxious chemical, osmotic and mechanical stimuli (Hilliard *et al.* 2005) and contribute to the avoidance of UV light (Liu *et al.* 2010) (Table 1). The PVD mechanoreceptors also contribute to proprioception (Cohen *et al.* 2012), and are activated by noxious cold (Chatzigeorgiou *et al.* 2010) (Table 1).

Future work will address several important challenges. An important unsolved issue is to obtain a biophysical understanding of sensory transduction. We do not yet understand how small temperature changes are sensed by the rGCs expressed in AFD to alter their enzymatic properties, nor is it clear how mechanical stress is transmitted across the skin to activate MeT channels in mechanoreceptor neurons. Another important issue is to decode whether the signaling pathways mediating responses to different sensory stimuli in a single sensory neuron type are compartmentalized according to the nature of the stimulus or whether they are integrated, enabling the neuron to deliver information regarding the coexistence of multiple sensory stimuli. Together with ongoing work on circuit computations in the *C. elegans* nervous system, a more detailed understanding of sensory transduction principles in this organism will allow us to derive general principles by which an animal navigates and thrives in its complex sensory environment.

## Appendix

### Laboratory Methods for Delivering Physical Stimuli to *C. elegans* Nematodes

The study of sensory transduction depends critically upon techniques for stimulus delivery. Below, we briefly review existing techniques for the delivery of physical stimuli (summarized in Table 2 and Table 3). We note that experimental designs often balance ease-of-use and quantitative precision.

**Table 2:** Mechanical stimulus delivery methods

	Tool or device*^a^*	Stimulus amplitude	References
Classical or qualitative	Eyebrow hair	Variable, 20–800 µN	Sulston *et al.* (1975), Chalfie and Sulston (1981), Nekimken *et al.* (2017b)
	Wire pick	Unknown	Way and Chalfie (1989)
	Plate tap	Unknown	Rankin and Broster (1992)
Quantitative approaches	Flexible glass probe (open loop)	Force, nN - µN	O’Hagan *et al.* (2005)
	Stiff glass probe (open loop)	Displacement, µm	Suzuki *et al.* (2003)
	Microcantilever (feedback control, closed-loop)	Displacement (µm) or force (nN-µN)	Park *et al.* (2007), Petzold *et al.* (2013), Eastwood *et al.* (2015)
	Pneumatic, microfluidic devices	Pressure-induced displacement (µm)	Cho *et al.* (2017), Nekimken *et al.* (2017a)

a See also Chalfie *et al.* (2014).

**Table 3:** Thermal stimulus delivery methods

	Tool or device*^a^*	Thermal range, gradient type	References
Spatial gradient	Frozen acetone	15° - ambient, Gaussian	Hedgecock and Russell (1975)
	Water bath plus metal substrate	Arbitrary, linear	Hedgecock and Russell (1975)
	Thermoelectric devices plus metal substrate	Arbitrary, linear	Ryu and Samuel (2002), Ramot *et al.* (2008b), Luo *et al.* (2014a)
Temporal variation	Substrate control		
	Superfusion	±10° from ambient, depending on thermal control device, slow (seconds)	Ramot *et al.* (2008a)
	Microfluidic device	±10° from ambient, depending on thermal control device, fast (sub-second)	Zariwala *et al.* (2003)
	Liquid droplet	±5° from ambient, slow (seconds)	Ryu and Samuel (2002), Clark *et al.* (2007b)

a See also Goodman *et al.* (2014).

#### Mechanical stress:

Mechanical stimuli are thought to generate stress within mechanoreceptor neurons and are generally reported as a function of the force applied, stimulator displacement, or the indentation produced in the tissue. The choice among these measures is primarily a matter of technical convenience and most tools operate in an open-loop control mode in which the output does not feed back onto the input. Classical tools for delivering mechanical stimuli to *C. elegans* are simple and hand operated (Chalfie *et al.* 2014)—an eyebrow hair glued to a toothpick or a platinum wire that doubles as a device for transferring worms between growth plates (Table 2). These tools are simple to use, but the amplitude, duration, timing, and location of stimulation are not under experimental control and are difficult to quantify. Moreover, the forces delivered by an eyebrow hair vary by an order of magnitude within and across human experimenters (Nekimken *et al.* 2017b). Fortunately, experimenters using an eyebrow hair tool consistently deliver forces that exceed the force required to saturate gentle touch responses (1–2 µN) by a factor of 5 (Petzold *et al.* 2013; Nekimken *et al.* 2017b; Mazzochette *et al.* 2018) (Table 2). Thus, the classical touch assay remains a valuable method for examining responses to touch in *C. elegans* nematodes.

The apparatus used for a plate tap withdrawal assay enables precise experimental control of stimulus timing and limited control over amplitude, but no control over stimulus location or duration (Table 2). An apparatus for plate tap accelerates a plunger into the side of a Petri dish to generate substrate displacements on the order of 10–80 µm with displacement amplitude depending on the strength of the plate tap (Timbers *et al.* 2013). Substrate displacement sufficient to evoke avoidance responses can also be achieved by using a piezoelectric sheet speaker to generate displacements up to 5 µm at frequencies between 0.5 and 3 kHz (Sugi *et al.* 2016).

The most common tool for controlling the amplitude, duration, timing, and location of mechanical stimulation is a piezoelectric motor pushing a glass probe into the animal’s body (Table 2). This general set-up is also used to study mechanosensitive ion channels in cells and typically operates without feedback control (Delmas *et al.* 2011). Although such open-loop systems offer good control over stimulus timing, duration, and position, their users must grapple with ambiguity regarding stimulus amplitude because it is not obvious how to determine the position of zero displacement or the indentation produced in response to a measured force or vice versa. Force-sensing devices provide this information and can be operated under feedback control to deliver a user-specified force or indentation. By contrast with open-loop systems, these closed-loop systems automatically regulate the applied force or indentation and two have been reported for use with *C. elegans* nematodes: one is based on custom-fabricated microforce-sensing probes (Park *et al.* 2007; Petzold *et al.* 2011, 2013; Eastwood *et al.* 2015) (Table 2) or other commercially available microforce sensing probes (Shaw *et al.* 2016; Elmi *et al.* 2017). Intracellular calcium responses generated in response to mechanical stimuli delivered by open-loop mechanical stimulators pushed into the ventral or dorsal side (TRNs, OLQ, CEP, FLP, PVD), down on the lateral aspect (ADL, ADE, PHB, ALA), or the tip of the nose (ASH) of animals immobilized by superglue are illustrated in Figure 2, A, B, and E, respectively. Closed-loop mechanical stimulators have yet to be applied in combination with calcium imaging.

Microfluidics-based devices for delivering mechanical stimuli to freely moving or immobilized *C. elegans* nematodes are emerging as an additional approach (reviewed by Kim *et al.* 2018, and summarized in Table 2). These include so-called artificial dirt chips that can test if *C. elegans* can distinguish between different textures (Han *et al.* 2017), as well as devices that deliver mechanical stimuli to immobilized animals (Cho *et al.* 2017; Nekimken *et al.* 2017a), or to animals allowed to move within defined channels (McClanahan *et al.* 2017). Because both the worms and the microfluidics chips are transparent, it is straightforward to combine these devices with calcium imaging, as illustrated in Figure 2, B and D.

#### Temperature:

Linear, spatial thermal gradients can be generated using several approaches, including thermoelectric devices or a temperature-controlled water bath coupled to a thermally conductive substrate such as an aluminum plate (Hedgecock and Russell 1975; Ryu and Samuel 2002; Yamada and Ohshima 2003; Ramot *et al.* 2008b) (Table 3). These gradients allow effective and efficient establishment of temperature gradients of the desired steepness and range. A glass substrate coated with indium tin oxide can be used to conduct thermal stimuli, and such a tool has been used in studies evaluating the response of thermosensory neurons to thermal gradients in restrained (Clark *et al.* 2006) and freely moving animals (Clark *et al.* 2007a). Strategies for controlled thermal variations in temperature include superfusion with temperature controlled saline (Ramot *et al.* 2008a; Wang *et al.* 2013), heating with an infrared laser (Wittenburg and Baumeister 1999; Clark *et al.* 2007b; Mohammadi *et al.* 2013), and rapid heating and cooling of droplets containing a single worm (Ryu and Samuel 2002) (Table 3). Microfluidic chips (Zariwala *et al.* 2003; McCormick *et al.* 2011) have also been developed for delivering acute thermal stimuli to restrained animals (Table 3). Additional details of these devices have been described recently (Goodman *et al.* 2014).

#### Others: magnetic fields, electric fields, light, humidity:

The study of responses to other physical stimuli including light, magnetic, and electrical fields, and relative humidity are in their infancy. Consequently, the tools for applying these stimuli have not yet been extensively refined. Current published methods for assessing responses to magnetic fields, electric fields, light, and humidity are described in brief below.

#### Magnetic and electrical fields:

Sensitivity to magnetic fields has been tested by exposing crawling animals to fields generated by a current-carrying coil surrounding a behavioral testing arena (Vidal-Gadea *et al.* 2015). Wild-type animals have been reported to be able to sense and respond to magnetic fields (Vidal-Gadea *et al.* 2015), although the inference that *C. elegans* can sense earth’s magnetic field has been disputed (Landler *et al.* 2018; Vidal-Gadea *et al.* 2018). To assess the response of *C. elegans* nematodes to electrical fields, animals are allowed to crawl on agar surfaces upon which a linear or rotating electrical field has been imposed (Sukul and Croll 1978; Gabel *et al.* 2007; Chrisman *et al.* 2016). Animals travel along remarkably straight paths toward the anode of a constant linear electrical field (Sukul and Croll 1978; Chrisman *et al.* 2016), and crawl in circles in response to a slowly rotating electrical field (Gabel *et al.* 2007). This behavior has been exploited in microfluidics devices to sort animals (Han *et al.* 2012; Wang *et al.* 2015).

#### Light:

To assess behavioral responses to light, pulses are delivered by coupling a shutter and a standard mercury bulb illuminator via an objective on a dissection microscope. To deliver light of specific wavelengths, the light source is filtered via standard microscope filters (Edwards *et al.* 2008; Liu *et al.* 2010; Bhatla and Horvitz 2015). Color-specific light-emitting diodes (LEDs) can also be used to deliver optical stimuli.

#### Humidity:

To establish a humidity gradient, distilled water or desiccant (such as DRIERITE(TM)) is placed into two troughs on either side of an elevated central stage in a plastic box. Worms are placed in the middle of a thin agarose pad on a glass plate placed on the central area, and the box sealed to establish the gradient (Russell and Pierce-Shimomura 2014). The gradient can be visualized using the colorimetric cobalt (II) chloride indicator.
